# FELASA Working Group report: Capture and transport of live cephalopods – recommendations for scientific purposes

**DOI:** 10.1177/00236772231176347

**Published:** 2023-11-03

**Authors:** Antonio V Sykes, Viola Galligioni, Juan Estefanell, Stuart Hetherington, Marco Brocca, Joao Correia, André Ferreira, Eleonora M. Pieroni, Graziano Fiorito

**Affiliations:** 1 CCMAR – Centro de Ciências do Mar do Algarve, Universidade do Algarve, Portugal; 2 Comparative Medicine Unit, Trinity College Dublin, Ireland; 3 Ciclo Superior Cultivos Acuicolas, Instituto de Educacion Secundaria les Profesor Cabrera Pérez, Spain; 4 CEFAS – Centre for Environment, Fisheries and Aquaculture Science, UK; 5 TECNIPLAST SpA, Italy; 6 Flying Sharks, Portugal; 7 Praceta do sol lote 4 n°57 3°D, Portugal; 8 Association for Cephalopod Research ‘CephRes’, Italy; 9 Department of Biology and Evolution of Marine Organisms, Stazione Zoologica Anton Dohrn, Italy

**Keywords:** Capture, transport, cephalopods, Directive 2010/63/EU, welfare, training

## Abstract

On 1 January 2013, research using cephalopod molluscs, from hatchlings to adults, became regulated within Directive 2010/63/EU. There are significant difficulties in captive breeding in the great majority of currently utilised species. Thus, scientific research relies upon the use of wild-caught animals. Furthermore, live cephalopods are shared and transported between different stakeholders and laboratories across Europe and other continents. Despite existing European and national legislation, codes, guidelines and reports from independent organisations, a set of recommendations specifically addressing the requirements for the capture and transport of animals belonging to this taxon are missing. In addition, although training and development of competence for all people involved in the supply chain are essential and aim to ensure that animals do not suffer from pain, distress or lasting harm, the requirements for those capturing and transporting wild cephalopods have not been considered. This Working Group reviewed the current literature to recognise scientific evidence and the best practice, and compiled a set of recommendations to provide guidance on the ‘techniques’ to be used for the capture and transport of live cephalopods for their use in scientific procedures. In addition, we propose to (a) develop standardised approaches able to assess recommended methods and objectively quantify the impact of these processes on animals’ health, welfare and stress response, and (b) design a training programme for people attaining the necessary competence for capture and transportation of live cephalopods, as required by Directive 2010/63/EU.

## Rapport du Groupe de travail de la FELASA. Capture et transport des céphalopodes vivants: recommandations à des fins scientifiques Résumé

Depuis le 1^er^ janvier 2013, la recherche sur les mollusques céphalopodes, des écloseries aux animaux adultes, est réglementée par la directive 2010/63/UE. L’élevage en captivité de la grande majorité des espèces actuellement utilisées présente des difficultés considérables; la recherche scientifique repose donc sur l’utilisation d’animaux capturés dans la nature. En outre, les céphalopodes vivants sont partagés et transportés entre les différents acteurs et laboratoires en Europe et sur les continents. Malgré les législations européennes et nationales, les codes, les lignes directrices et les rapports des organisations indépendantes, il manque un ensemble de recommandations traitant spécifiquement des exigences de capture et de transport des animaux appartenant à ce taxon. En outre, bien que la formation et le développement des compétences de toutes les personnes impliquées dans la chaîne d’approvisionnement soient essentiels et visent à s’assurer que les animaux ne souffrent pas, ne soient pas en détresse ou ne subissent pas de dommages durables, les exigences liées à la capture et au transport des céphalopodes sauvages n’ont pas été prises en compte. Ce groupe de travail a examiné la littérature actuelle pour en tirer les meilleures pratiques et les preuves scientifiques, et a compilé un ensemble de recommandations fournissant des orientations sur les «techniques» à utiliser pour la capture et le transport des céphalopodes vivants afin de les utiliser dans des expériences scientifiques. Des efforts futurs sont également proposés pour (a) développer des approches normalisées capables d’évaluer les méthodes recommandées et de quantifier objectivement l’impact de ces processus sur la santé des animaux et la réponse au stress; (b) concevoir un programme de formation permettant d’atteindre les compétences nécessaires pour la capture et le transport des céphalopodes vivants, conformément à la directive 2010/63/UE.

## Fang und Transport von lebenden Kopffüßern: Empfehlungen für wissenschaftliche Zwecke – Bericht der FELASA-Arbeitsgruppe Abstract

Seit dem 1. Januar 2013 ist die Forschung mit Kopffüßern, vom Schlüpfling bis zum ausgewachsenen Tier, durch die Richtlinie 2010/63/EU geregelt. Bei der großen Mehrheit der derzeit genutzten Arten gibt es erhebliche Schwierigkeiten bei der Zucht in Gefangenschaft, sodass die wissenschaftliche Forschung auf die Verwendung von Wildfängen angewiesen ist. Außerdem werden lebende Kopffüßer zwischen verschiedenen Beteiligten und Labors in ganz Europa und auf allen Kontinenten ausgetauscht und transportiert. Trotz der bestehenden europäischen und nationalen Rechtsvorschriften, Kodizes, Leitlinien und Berichte unabhängiger Organisationen fehlt es an Empfehlungen, die sich speziell mit den Bedingungen für den Fang und Transport von Tieren dieses Taxons befassen. Zudem finden die Voraussetzungen für Personen, die wilde Kopffüßer fangen und transportieren, bisher keine nähere Berücksichtigung, obwohl die Ausbildung und die Entwicklung von Kompetenzen für alle an der Beschaffungskette beteiligten Personen von wesentlicher Bedeutung sind und um sicherzustellen, dass die Tiere keine Schmerzen, Ängste oder dauerhaften Schäden erleiden. Diese Arbeitsgruppe hat die diesbezügliche aktuelle Literatur gesichtet, um die besten Praktiken und wissenschaftlichen Erkenntnisse zu ermitteln, und eine Reihe von Empfehlungen zusammengestellt, um eine Anleitung für die Methoden zu geben, die für Fang und Transport von lebenden, zur Verwendung in wissenschaftlichen Verfahren bestimmten Kopffüßern genutzt werden sollten. Darüber hinaus werden Maßnahmen für die Zukunft vorgeschlagen, um (a) standardisierte Ansätze zu entwickeln, die es ermöglichen, die empfohlenen Methoden zu bewerten und die Auswirkungen dieser Prozesse auf die Gesundheit und die Stressreaktion der Tiere objektiv zu quantifizieren; (b) ein Schulungsprogramm für Personen zur Erlangung der erforderlichen Kompetenz für den Fang und Transport lebender Kopffüßer zu entwickeln, wie in der Richtlinie 2010/63/EU gefordert.

## Informe de FELASA Working Group Captura y transporte de cefalópodos vivos: recomendaciones para fines científicos Resumen

El 1 de enero de 2013, la investigación con moluscos cefalópodos, desde crías hasta adultos, pasó a estar regulada por la Directiva 2010/63/UE. Existen complicaciones significativas para la cría en cautividad en la gran mayoría de las especies utilizadas actualmente, por lo que la investigación científica se basa en el uso de animales capturados en la naturaleza. Asimismo, los cefalópodos vivos se comparten y transportan entre diferentes partes interesadas y laboratorios de toda Europa y en otros continentes. A pesar de la existencia de legislaciones europeas y nacionales, códigos, directrices e informes de organizaciones independientes, falta un conjunto de recomendaciones que aborden específicamente los requisitos para la captura y el transporte de animales pertenecientes a este taxón. También cabe destacar que, aunque la formación y el desarrollo de la competencia de todas las personas implicadas en la cadena de suministro son esenciales y tienen como objetivo garantizar que los animales no sufran dolor, angustia o daños duraderos, no se han tenido en cuenta los requisitos para quienes capturan y transportan cefalópodos salvajes. Este grupo de trabajo revisó la bibliografía actual para reconocer las mejores prácticas y pruebas científicas, y recopiló un conjunto de recomendaciones para proporcionar orientación sobre las ‘técnicas’ que deben utilizarse para la captura y el transporte de cefalópodos vivos para su uso en procedimientos científicos. Asimismo, se proponen esfuerzos futuros para (a) crear métodos estandarizados capaces de evaluar los métodos recomendados y cuantificar objetivamente el impacto de estos procesos en la salud y la respuesta al estrés de los animales; (b) diseñar un programa de formación para que las personas adquieran la competencia necesaria para la captura y el transporte de cefalópodos vivos, con arreglo a la Directiva 2010/63/UE.

## Introduction

Cephalopods (nautilus, cuttlefish, sepiolid, squid and octopus) are the sole invertebrates listed among the species regulated by Directive 2010/63/EU for use in scientific research. The taxon counts about 800 living species, all marine, and constitutes a class belonging to the phylum *Mollusca*. Over the last decades, discoveries about cephalopod biological, evolutionary, morphological, genomic and physiological innovations and adaptations, as well as their neural and cognitive characteristics, have promoted a renewed interest for these animals, favouring the increase in the number of studies and species utilised for scientific purposes.^1–6^ At the same time, the relevance of their welfare status^7,8^ and its consequences on the scientific outcome have increased in both the commercial and the scientific fields.

Article 9 of Directive 2010/63/EU specifies that animals must not be taken from the wild for use in procedures (Article 9.1) unless the relevant National Competent Authority (NCA) grants an exemption (Article 9.2) based on the scientific justification that the purpose cannot be achieved using bred animals.^9^

The Directive also requires that the capture of live wild animals should be accomplished by competent people using methods which do not cause avoidable pain, suffering, distress or lasting harm (PSDLH; Article 9.3). In addition, adequate care must be taken to prevent physical injury and stress to the animals at all stages in the supply chain, including capture, transportation and acclimatisation to laboratory conditions (and quarantine when required). Consequently, the capture and transportation of animals from the wild should be well planned, meticulously prepared and effectively performed.

For the vast majority of laboratory animals – including vertebrate aquatic model species – production technology has reached a maturity that allows their breeding for use in procedures. On the contrary, cephalopod culture is still in its infancy, having faced several bottlenecks to the point that few cephalopod species are currently cultured in captivity at a limited local scale.^10,11^ Culture protocols of cephalopods for scientific purpose are not fully developed yet, and evidence for successful rearing of multiple generations in captivity^12–14^ without altering their welfare and behaviour is still lacking, possibly with a few exceptions. Similarly, doubts and criticisms arise around the development of a possible industrial aquaculture for these animals^15,16^ considering their sentience, sophisticated neural organisation and cognitive capabilities.^1–3,7^

Despite the increased scientific interest in these organisms, most of the research performed on these molluscs still relies upon the collection and transport of wild-caught animals. Here, we present the outcomes of the work of the FELASA Working Group ‘Capture and transport of cephalopods’ with the aim of developing recommendations about methods to be utilised and guidance about the required competence of people involved in the capture and transport of these animals for scientific research.

## Cephalopods as laboratory animals: a legislative framework for capture and transport

Since Directive 2010/63/EU came into force in 2013, no gold standard method has been proposed to capture wild aquatic animals – including cephalopods – for use in scientific research. We analysed the available legislation and recommendations regarding the capture and transport of aquatic animals, and we identified several regulatory and recommendation documents of interest. A summary of the outcomes of our analysis is available in Suppl_1:(Legislative framework in the Supplemental Material), while a detailed review is provided in the ancillary work.^17^

From the analysis any legislative or regulatory document with explicit mention of cephalopods were found. In addition, we found that most of the recommendations are aimed at protecting animals during transportation, with limited detail provided for the capture from the wild, at least when aquatic species are considered.

Another key and reiterated element in these recommendations is the attention given to the competence of the personnel, that is, the need for proper training for those involved in the capture and transport of wild animals. All of the recommendations point out that the main source of suffering and distress in animals being captured, handled and transported relates to the limited competence of the people involved.

Despite the existence of European Directives and various guidelines and documents (see Suppl_1: Legislative framework in the Supplemental Material, and Pieroni et al.^17^), it is clear that regulations on capture and transport lack specific recommendations about wild cephalopod species, at both general and species-specific levels (for a detailed overview, see table 1 in Pieroni et al.^17^). However, considerations and precautions on capture and transport of cephalopods for research purposes are included in the Guidelines for the Care and Welfare of Cephalopods in Research.^18^

**Table 1.** Capture methods.

Capture method	Nautilus	Cuttlefish	Sepiolidae	Squid	Octopus
Trawl	NRW, E	NRW, E	NRW, E	NRW, E	NRW, E
Traps	**BLT** Juv, Ad GPS	**BkT, CtT** SzS, Ad	NRSzI	***BLT*** SzS, Ad GPS	***JBP*** SzS, Juv, Ad GPS
Pots	Not adopted	Not Adopted	NRSzI	***JBP*** SzS, Juv, Ad, GPS	**Pt, JBP** Juv, Ad
Jigs	Not Adopted	NRW, E	Not Adopted	NRW, E	NRW, E
Nets	Not Adopted	***TN*** Juv, Ad	**DN, SN** Juv, Ad	**PN, SN**, Ad**BN**Hatch	Not AdoptedHatch

Overview of the most and least recommended capture methods of live cephalopods for research purposes. Data are summarised by method (i.e. trawl, traps, pots, jigs and nets) and taxon. For a detailed review, see ancillary work of Pieroni et al.^17^ and table 2 therein. Recommendations are coded: boldface for the most recommended method; bodface and italics for the second most recommended method; Not Adopted for not generally adopted method; NR, not recommended.

Ad: adult; Bkt: basket traps; BLT: baited or light traps; BN: bongo nets; CtT: cuttlefish traps; DN: dipnets; E: environmental issue; GPS: global positioning system; Hatch: hatchling; JBP: Japanese baited pot; Juv: juvenile; PN: pound nets; Pt: pot; SN: seine nets; SzI: size-issue; SzS: size-selective; TN: trammel nets; W: welfare issue. For taxon-specific details, see Suppl_4 in the Supplemental Material.

## Recommendations for capture and transport of cephalopods in research

The purpose of this Working Group was to carry out detailed text mining, which identified more than 100 published works. This informed a review of existing capture methods for collecting and about those adopted in transporting live wild cephalopods. The outcome of this analysis is not provided herein but is presented in detail in the ancillary work.^2^

The analysis of the available information highlighted important considerations:

- All the capture methods reported in various studies have been taken from fishery and readapted in a small set of cases for scientific purposes.
- There are no species-specific procedures but rather several ‘protocols’ and/or variants for the same method.
- No particular attention is given to the different life stages of cephalopods used in the studies, and this piece of information is often missing.
- Very little information is provided about the capture and transport procedures adopted, and in most cases, only one of the two ‘parts of the story’ is described.
- Some papers provide a list of recommendations which are mainly anecdotal or derive from indirect communications, and they should therefore be validated further by robust studies.

### Maximising welfare during the capture of live wild cephalopods

According to the General Section 4.2 of the Council Recommendation of 18/06/2007:

- Animals should be captured by ‘humane methods and by persons competent to apply them’, minimising ‘the impact of the capturing procedures on the remaining wildlife and habitats’.
- ‘Any animal found to be injured or in poor health should be examined by a competent person … In case of serious injury, the animal should be killed immediately by a humane method’ described in the Directive 2010/63/EU.
- ‘Appropriate and sufficient transport containers and means of transport should be available at capture sites, in case animals need to be moved for examination or treatment’.^19^

How should we apply all of this advice to cephalopods? There is not a unique reliable method to capture every cephalopod species, but rather a small range of techniques that best fits the species-specific needs, also considering their life stage, physiology and inter-individual variability. When considering the following capture methods (Table 1), we recommend associating a severity assessment in order to try to predict the impact of a given protocol on the welfare of wild cephalopods.

### Fishing: what can we learn from it?

Cephalopods are animals of great interest for commercial purposes, currently accounting for 5% of the marine capture volume worldwide,^20^ with a significant increase in demand, although this was recently slowed by the COVID-19 outbreak (see Suppl_RN: Reference to the ancillary work and other notes in the Supplemental Material). For this reason, a great number of capture methods have been developed by artisan and small-scale fisheries.^21^ On the other hand, many reports on cephalopods biology and fishing have been published (for a detailed review, see table 2 in Pieroni et al.^17^), but no comprehensive summary of the numerous cephalopod capture methods is currently available for a given species and life stage. However, Rathjen^22^ stated the need for ‘more resource-friendly’ fishing methods for these animals. In his work, line jigging was the most suitable fishing gear for squids (*Loligo forbesii*, *Illex argentinus*, *Todarodes pacificus* and *Nototodarus sloanii*). The technique appears selective and adjustable for the size of the specimen, thus limiting the impact on the environment or other fauna. Trawling is also much utilised for fishing cephalopods, but this results in the by-catch of other animals, as it is not species or size specific.

**Table 2.** Transport methods.

Transport method	Nautilus	Cuttlefish	Sepiolidae	Squid	Octopus
Plastic bag	NR	**M** Juv, Ad S > 12 hours	**M** Juv, Ad S > 21 hours	**I** S > 20 hours	Hatch, MS (6, 12, 24 hours)Ad, IS (8–12 hours)
Box	**M**; ChW Juv, Ad S (4–12 hours)	***ChB*** Juv, Ad Sub	***Chb*** Juv, Ad Sub	***Bu***, ***Co***, Ba, I Juv, Ad S (8–11 hours)	***Tu***, ***J***, ***Cr***; ***Chb***, I (for: Juv, Ad) S (>24 hours)
Tank	***Chb*** Juv, Ad Sub	Chb Juv, Ad Sub	Not AdoptedChbJuv, Ad	Not AdoptedLSz; ChbJuv, Ad	**I** Juv, Ad S > 12 hours

Overview of the most and least recommended transport methods of live cephalopods for research purposes. Data are summarised by method (container; i.e. plastic bag, box, tank) and taxon. For a detailed review, see ancillary work of Pieroni et al.^17^ and table 3 therein. Recommendations are coded: bodlface for the most recommended method; bodface and italics for the second most recommended method; underlined for the third most recommended method; Not Adopted, not generally adopted method; NR, not recommended.

Ad: adult; Ba: barrel; Bu: buckets; Chb: containers for holding bags; ChW: chilled water; Co: coolers; Cr: creels; Hatch: hatchling; I: individual storing; LSz: large sample size; J: jars; Juv: juvenile; M: multiple storing possible; S: survival; Sub: substrate; Tu: tube. For taxon-specific details, see Suppl_4 in the Supplemental Material.

Traps and pots are utilised in many geographical areas and represent traditional gears for fishing cephalopods. These rely on the natural trend of some species to search for dens and hidden refuges. Spearing, multiple hooks and trolling are still used, although with local adaptions and relatively small variations.

In our opinion, the fundamental question for the scientific community would be whether we can adjust some of the currently available fishing methods to render them suitable for collecting cephalopods used for research purposes. The answer is yes, providing that coordinated, cooperative interaction between different stakeholders is established.

### Capture of live cephalopods for research purposes

An overview of the best recommended methods for the capture of live cephalopods for research purposes is provided in Table 1.

#### Nautiluses

Collectors and scientists unanimously agree upon the use of baited or light traps as the best method for capturing live wild nautiluses. A prototype originally reported by Carlson^23^ is still utilised with some variations, including monitoring systems.

#### Cuttlefishes and sepiolids

From the little information available about the capture methods for cuttlefishes, the most feasible technique seem to be traps, in particular basket or cuttlefish traps, very similar to those employed for squid but larger and lighter. Under these conditions, juvenile and adult cuttlefishes are captured uninjured. These size-selective gears may allow for the addition of the seabed which can be used as an attractive spawning substrate for broodstock destined for aquaculture purposes.^24^ Large nets, such as trammel nets, are also suitable for catching both juvenile and adult animals without excessive constraint and without resulting in any environmental issue as is the case for trawling. Seine nets and dipnets are mostly used for adult forms of sepiolids (e.g. *Euprymna scolopes* and *E. tasmanica*) destined for research and are considered the less traumatic method for these small cephalopods.^25^

#### Squids

For squids, one of the most frequently employed capture method is the jig lure with barbless hooks operated mechanically or by hand.^26^ Analyses of the impact of this capture method revealed that it induces some injury to the animals (see Table 1 and Suppl_4: Supporting info to the overview of capture and transport methods in the Supplemental Material). Alternatively, several kinds of nets have been utilised to capture squids for laboratory use: pound nets, bongo nets, seine and dip nets which all proved to be harmless. These nets are large, and squids are able to swim before they get caught, forming a consistent sample size.^17,27^ Furthermore, these appear suitable for capturing specimens of any life form, but attention must be paid to the by-catch of egg masses.

#### Octopuses

Undoubtedly the best existing capture method for octopuses is the pot.^21,28,29^ Pots, like traps, are generally made of natural, non-toxic materials with non-abrasive surfaces and exploit the natural tendency of these animals to search for a den. Octopuses spontaneously settle in these gears which are very likely to catch undamaged specimens. As reviewed,^17^ pots should have a dark tone, narrow entrance and large interior that allows the animal to settle and see outside without exposing itself to danger. A series of adjustments have been proposed, such as the insertion of a GPS monitoring system or the addition of a removable lid that might also be useful for transportation. Pots are alluring for both juveniles and adults, and very often they can be chosen by females as substrate for laying eggs, which, if not required for the aim of the project, should be returned to nature afterwards. A combination between pots and traps are the so-called Japanese baited pots,^30^ combining shelter and bait (Table 1).

### Transport methods of cephalopods: maximising welfare from sea to the lab and between labs

With regard to capture, the best transportation methods should avoid (or at least limit) PSDLH and should not increase the stress associated with the capture technique. Again, most likely, there is not a unique protocol to transport cephalopod species (for a detailed review, see table 3 in Pieroni et al.^17^); inter-individual variability and species-specific features (e.g. body size, physiology and biological requirements for every life stage) must be considered when preparing the animal for the journey.

A detailed list of considerations for transportation of live cephalopods for research purposes is provided by Fiorito and colleagues^18^ which in turn is mostly based on available guidelines for the transportation of live fishes,^31^ in compliance with the codes and regulations for European and international transport of live animals (see Suppl_1.2: Principles for transport, documentation and planning of the journey in the Supplemental Material). The work could be therefore considered as the groundwork for building upon the best recommendations for cephalopod transport summarised here in Table 2.

In the first known guidelines on the rearing of cephalopods for scientific use (Grimpe’s ‘Care, treatment and rearing of cephalopods for zoological and physiological purposes’),^32,33^ several insights about the methods of transportation and maintenance of the animals during the journey for different species of cephalopods are provided. These can be considered for the development of good practice. In Grimpe’s words, a vital aspect is always to keep cephalopods in well-oxygenated seawater throughout the journey, as these animals have a high metabolic rate that rapidly produces large amount of carbon dioxide and ammonia.^34–37^ For this reason and to limit distress (e.g. agonistic attacks and aggression, inking), animals should be kept individually in separate bags. Some cephalopods are shipped in Styrofoam fish boxes (cooled or heated according to the species), although a temperature slightly below the optimum has been suggested because it reduces the animals’ metabolic rate, allowing the shipping water to hold more oxygen and reduce waste production.^10^ However, containers should be kept in the shade when being transporting (e.g. by boats), or air conditioning should be used when transporting by car or other vehicles (see Suppl_3: Other recommendations for transport of live cephalopods in the Supplemental Material).

Currently, there are no specific aerated containers designed for cephalopods, and neither is there an open system for their transport, but these could be obtained by adjusting those available for live fish transportation.^31,38^ Containers can be provided with substrate only during short journeys, as the addition of organic material can be subjected to decomposition processes, thus reducing water quality and oxygen, potentially compromising animal welfare during longer journeys; sand poses the risk of H_2_S release from fouled sediments. Sedation (e.g. cold water, MgSO_4_ or MgCl_2_) is not essential and is not recommended for the transport of most cephalopods, despite being suggested in some cases.^39^

With regard to capture methods, we reviewed the literature concerning the transportation of cephalopods (see Pieroni et al.^17^ and table 3 therein) in order to extrapolate general indications that might be useful for creating some species-specific recommendations. Here, we summarise the recommended methods for the transport of live cephalopods for research purposes (see also Table 2).

#### Nautiluses

Boxes or insulated chests with chilled seawater have been recommended for both juveniles and adults of different species of nautiluses and are preferred to plastic bags, as these can wear out, putting the welfare of the animals at risk. More specimens can be contained in the same box, providing each animal with at least 4 L of seawater.^23^

#### Cuttlefishes and sepiolids

The transportation of cuttlefish can be challenging; animals should be transported in plastic bags or barrels, using large containers to store them. It has been suggested to transport fewer animals per plastic bag or barrel according to the size.^40^ Our recommendation is to transport animals individually and not as ‘group’ of individuals. As for cuttlefishes, sepiolids are mainly transported in plastic bags containing few individuals according to body size and the relative final volume of the container. It is recommended to put these bags in larger insulated boxes that ensure no leakage or asphyxiation of the animals.^13^

#### Squids

During transport, squids should be individually placed in plastic bags (preferably laid horizontally), barrels or buckets filled with seawater and oxygen in appropriate proportions (Table 2), sealed and placed in larger tanks or Styrofoam boxes.

#### Octopuses

The personal experience of Grimpe^32^ with *Octopus vulgaris, Eledone moschata* and other species made the author suggest the use of enamel pots placed in Demijohn baskets with stuffed hay between them in order to reduce potential insults related to the transport method.^33^ These have a cylindrical base and are conically tapered at the top. Only the lower part of the pot (containing between 20 and 80 L, depending on the size of the Demijohn) should be filled with seawater; the rest must be air, the circulation of which must be assured by multiple holes in the cork.^32^ Pots, such as those utilised for the capture of octopuses, may be employed to facilitate transportation (see Table 2). These should be placed in a larger container or tank, as in a modern version of Demijohn baskets. Containers must be filled with seawater (recommended from the collection site) saturated with oxygen. Our suggestion based on knowledge of the biology of these animals is to keep each in individual separate bags or pots and not together with other specimens. Grimpe’s original recommendation^32,33^ has been readapted in different ways, but his approach for successful transport of live cephalopods is still valid and has been widely applied.

## Eggs as a possible alternative

The collection and standardised transport of eggs for target cephalopod species have been proposed as an alternative, since these appear easier to manage. For ‘classic’ laboratory species (e.g. fish), moving embryos, sperm or eggs between research facilities has increased and is now well established. This approach is also considered as a way to comply with one of the 3Rs – Refinement – since transport appears to be stressful for live animals and could impact their welfare, while for early life stages, it seems to have limited effects (although no specific studies are known). On the other hand, the use of captive animals raised from eggs would allow experimental requirements to be fulfilled, such as controlling previous experiences.

With regard to cephalopods, consideration should be given to offer a standardised method of egg collection to reduce the impact on natural resources (e.g. collecting those stranded on the coastline or those originating from by-catch) and to minimise the harm to the egg masses. By-catch as a source of eggs should be the recommended option. Of course, this is not possible for all species currently used in research. Although cephalopod eggs need to be handled delicately, accurate temperature and salinity control during all phases of the process, including transportation, are equally important.^41,42^

In terms of eggs, it is preferable to obtain egg clutches in the middle stage of development because the survival rate is the highest, whereas during the early stages, death of the embryos is more likely to occur. On the other hand, eggs collected at advanced stages of development may prematurely hatch during transport, as their metabolism is very high,^43^ and such conditions should therefore be attentively taken into account to fulfil any welfare requirement properly.

For some cephalopod species, maternal care is needed (e.g. incirrate octopods or oceanic squids^44,45^), and attempts to simulate properly are mandatory. Maternal care is a critical factor for proper embryonic development and for limiting the risk of premature hatching.^10,46–49^ The utmost attention is required in these cases, and specific standardised protocols require further development, despite some attempts.^32,42,50^

Directive 2010/63/EU considers the protection of this taxon from hatching. However, several studies are based on the collection and culture of egg masses from the wild which – although easier to obtain and then transport in terms of the size of containers and water volume – require particular care, being very sensitive even to small changes in temperature, pH and salinity.^51^ Storing conditions during embryonic development should also be monitored because the health state of the eggs will consequently affect the ‘quality’ of the hatchlings.

Methods for transport of eggs of several cephalopod species have been developed, achieving some standardisation; examples are available for cuttlefish, squid and octopus eggs.^32,40,42^

## Future needs and how to achieve them

Two main actions are suggested for the future: (a) collaboration between fishermen and scientific research to standardise and implement further the best recommended methods for the capture and transport of live cephalopods for research purposes; and (b) a training programme that may help to increase the acquisition of the required competence for the people involved. Here, we briefly discuss these two actions.

### Developing an experimental approach to validate recommended methods

What emerges from the analysis of the scientific literature, various recommendations, technical reports and unpublished data (for a review, see ancillary work^17^) is the need for more in-depth studies on the capture and transportation methods that are specifically able to address the best way to handle these animals and their welfare under such circumstances. Body size, life stages and the biological and physiological needs of the species are all fundamental aspects to consider. The vast majority of methods currently utilised are based on personal experience and interaction with local fishermen, but relatively little scientific systematic studies have been carried out for the purpose of assessing the best capture and transport methods for cephalopods.

Approaches to assess the stress response (indicator of animal welfare) of aquatic animals to the capture and transport methods have been applied to fish and a few crustaceans, supporting the identification of the most appropriate catching gear to be used for these animals. These studies also provide useful approaches for the design of taxon-specific containers suitable for different modalities of transportation (for a review, see Pieroni et al.^17^). With regard to cephalopods, only recently, Araújo and co-workers^52^ studied the effects of a simulated long-journey transportation at high density on live *Octopus vulgaris*. No mortality was recorded at the end of 48 hours of transportation at the different densities of animals considered, and no significant changes in the physiological parameters were found. In another study, Barragán-Méndez and colleagues^53^ evaluated the impact of some octopus species after capture (i.e. trawl; see also Pieroni et al.^17^). Despite these works, studies are still required to facilitate informed guidance on species-specific capture and transport methods based on the assessment and control of stress-induced levels in live cephalopods. This Working Group promotes ad hoc studies that will help to achieve this goal.

We recommend a set of experiments designed to evaluate the physiological effects of different combinations of capture and transport methods on both sexes of juveniles and adults of the most commonly utilised cephalopod species in scientific research. The idea behind this is to adopt a collaborative effort with selected geographically distributed fishermen communities using a significant number of individuals for each species (and possibly in two separate seasons), utilising a couple of capture methods chosen for comparison with those claimed to be the most recommended ones such as: (a) nets versus traps in cuttlefishes, (b) jigs versus traps in squids and (c) pots versus traps in octopus. Target species should be those mostly utilised in research to facilitate the focus of these studies. Following capture, different transport conditions will be tested (e.g. individual vs. multiple ‘storing’ of animals kept in standard, open, large, darkened buckets or plastic bags). After the transportation phase (3–4 or 24 hours, testing duration of transport), the welfare of the animals will be assessed using a panel of indicators selected among those included in table 5 of the FELASA guidelines.^18^ Monitoring individual animals should be performed (a) immediately after capture, (b) immediately after transportation and (c) on day 1 and on day 4 or 5 after capture to measure how much time the animals take to recover and acclimatise to the estimated baseline levels of these physiological indicators (for additional details, see also Pieroni et al.^17^). Once preliminary studies have proven effective in guaranteeing the survival of the cephalopods, other investigations will follow in close collaboration with fishermen to reach a consensus on the benefits and expanding the study to a larger group and conditions (geographical, boats, etc.).

We aim to find the best conditions for capture and transport that could be used to improve animal welfare in different circumstances (e.g. capture and transport of different life stages, intercontinental journeys, etc.). In addition, we are convinced of the importance of the active involvement of fishermen and transporters as scientific suppliers for live cephalopod species.

### Increasing competence and good practices: towards ad hoc training

Article 23 of Directive 2010/63/EU specifies the need for competent personnel when (a) carrying out procedures on animals, (b) designing procedures and projects, (c) taking care of the animals and (d) killing animals in order to limit and/or avoid the induction of PSDLH in the animals.^9^ Both the capture and transport of cephalopods are part of the phases that relate to the ‘life’ of live animals for scientific purposes and must be performed by trained and expert personnel. As such, in our view, the ‘working document on the development of a common education and training framework to fulfil the requirements under the Directive’ should be addressed also to people specifically involved in the capture and transport of living cephalopods. Annex IV of EU Council Regulation No 1/2005 has already provided instructions concerning the training for transporters which shall include notions on animal physiology and their needs, handling and impact on stress and welfare.^54^

Here, we propose a training programme (see Suppl_Box 1 in the Supplemental Material) designed for the suppliers of live animals to improve their competence in dealing with cephalopods with the aim of assuring compliance with species-specific biological, physiological and behavioural needs and welfare requirements.

We are convinced that by focusing on the training of fishermen – whose expertise and practical knowledge of the sea are undoubtable – we will be able to develop the best practice for capturing cephalopods in the most humane way. Of course, fishermen’s beliefs and behaviour depend upon the economic and social structure within which they are operating (see discussion in Pieroni et al.^17^). We want to rely firmly on the experience of proud lifetime cephalopod artisan and small-scale fishers, and we are interested in their holistic analysis of the context. What we want to achieve is joint action between trained personnel, which will ensure that wild-caught cephalopods will be properly captured and transferred to their destination without experiencing unacceptable pain or suffering.

The most challenging part is to approach fishermen and transporters and persuade them to take part in the training and education process. The biggest resistance could be due to the loss of potential work by undertaking the training without having beneficial personal profit. It is therefore our aim to find a general balance between their needs and the obligations necessary for fulfilling competence, as required by the increasing attention on all aspects of animal care due to the inclusion of live cephalopods in the regulations for scientific research purposes. In our view, incentives should be provided for attendees who will partake in becoming suppliers of live wild animals for the laboratories, such as gaining a greater income and the possibility of working throughout Europe and not necessarily just in their own country or for the local supply. Moreover, since the successful trainees will receive a certificate of completion, this may help them to access fishing licenses more easily according to national (and possibly international) legislation. A coordinated effort between different stakeholders, including non-profit organisations and local and national governments, will then be required. The training framework should be accessible, affordable and – with joint effort from the member states – hopefully free, as well as being flexible to meet the working time or shifts of the trainees.

Suppl_Box 1 in the Supplemental Material summarises the organisation of the proposed training programme. It is our aim that fishermen and transporters of live cephalopods should be actively encouraged to face the challenge of improving the well-being of the animals they work with, by achieving awareness about the concept of welfare, the biological needs of the species and ethical approaches when dealing with cephalopods as animals destinated for scientific work. In order to reach the maximum degree of commitment, a special version of a dedicated training course for cephalopods will be designed so as to allow people coming from different cultural backgrounds to undertake an initial induction course that will help them to reach the same level when approaching the objectives of the main training programme (see Suppl_Box 1 in the Supplemental Material). Once successful, collectors and shippers may start working under the supervision of an expert group for a period of time to monitor further if the required skills match the animals’ and stakeholders’ benefits, and to assure animal welfare will be always the top priority.

## Concluding remarks

In this report, the Capture and Transport of Cephalopods FELASA Working Group wanted to highlight the current limited knowledge about protocols for the capture and transport of cephalopods destined for scientific research. European legislation and recommendations are not sufficient and do not directly address cephalopods (see the review in Pieroni et al.^17^), while regulations concerning the wildlife capture methods are poorer in taxon-specific information, even for vertebrates. Different organisations and few other countries include cephalopods among the animals whose welfare should be protected during the capture and transport and are mainly bound to international transport and shipping rules. From the text mining carried out in this Working Group (see also the ancillary work^17^), some considerations emerged about the need never to omit fundamental information in scientific work, as also recommended by PREPARE^55,56^ and ARRIVE^57,58^ guidelines. We attempted to define the capture and transport conditions likely to be the most suitable for specific taxon (Tables 1 and 2) with attention to the target life stages needed for experimental studies (see Pieroni et al.^17^).

General considerations that can be drawn from this analysis are:

1. The best capture method is any harmless tool that exploits the natural behavioural tendency of the animal (e.g. seabed substrate for spawning cuttlefish, octopus’s preference for dens) and that considers its daily cycle and diet composition – according to the species and its life stage – to catch it more efficiently. A good capture method should be classified as a mild procedure, and therefore the target cephalopod should experience only short-term distress. All the large-scale non-selective methods (e.g. trawl) must be avoided because of the impact on the welfare of the animals (and on the environment^59–62^).
2. The best transportation method is the one able to avoid or reduce further stress related to the capture procedure. The key factor in preventing the animals from experiencing PSDLH is planning (duration, resting place and number of health checks), thus avoiding any factor that may compromise animal welfare. Environmental requirements (e.g. oxygen, pH, salinity and temperature) must be monitored throughout the journey, and they should fit the welfare requirements of the different cephalopod taxa. Depending on the duration of the journey, particular attention should be paid to the type, size and equipment of the means of transport, as well as the containers used while on board.

These two processes are not independent from each other. To transport the animals better, the capture procedure should be done properly to avoid any handling and exposure to aversive conditions from the collecting site to the container. With this in mind, we propose pilot studies that could be carried out in a collaborative fashion with select geographically distributed fishermen communities to compare the effect of different combinations of capture and/or transport methods on the survival rate, physical conditions and physiological milieu of the captured (and transported) individuals belonging to the most common cephalopod species (e.g. cuttlefish and octopus).

Finally, the competence of the personnel carrying out these activities is pivotal in the success of both capture and transport methods. For this reason, we propose a special version of the education and training programme for cephalopods (CBC FELASA accredited training programme) dedicated to fishermen and transporters and designed to consolidate the required competence and attention to animal welfare.

## Supplemental Material

sj-pdf-1-lan-10.1177_00236772231176347 - Supplemental material for FELASA Working Group report: Capture and transport of live cephalopods – recommendations for scientific purposes Supplemental material, sj-pdf-1-lan-10.1177_00236772231176347 for FELASA Working Group report: Capture and transport of live cephalopods – recommendations for scientific purposes by Antonio V Sykes, Viola Galligioni, Juan Estefanell, Stuart Hetherington, Marco Brocca, Joao Correia, André Ferreira, Eleonora M. Pieroni and Graziano Fiorito in Laboratory Animals

## References

[1] Marini G De Sio F Ponte G , et al. Behavioral analysis of learning and memory in cephalopods. In: Byrne JH (ed) Learning and memory: a comprehensive reference. 2nd ed. Amsterdam: Academic Press, Elsevier, 2017, pp.441–462.

[bibr2-00236772231176347] Ponte G Chiandetti C Edelman DB , et al. Cephalopod behavior: from neural plasticity to consciousness. Front Syst Neurosci 2022; 15: 787139. 35495582 10.3389/fnsys.2021.787139 PMC9039538

[bibr3-00236772231176347] Edelman DB Seth AK. Animal consciousness: a synthetic approach. Trends Neurosci 2009; 32: 476–484. 19716185 10.1016/j.tins.2009.05.008

[bibr4-00236772231176347] Albertin CB Simakov O. Cephalopod biology: at the intersection between genomic and organismal novelties. Annu Rev Anim Biosci 2020; 8: 71–90. 31815522 10.1146/annurev-animal-021419-083609

[bibr5-00236772231176347] Liscovitch-Brauer N Alon S Porath HT , et al. Trade-off between transcriptome plasticity and genome evolution in cephalopods. Cell 2017; 169: 191–202. 28388405 10.1016/j.cell.2017.03.025 PMC5499236

[6] Albertin CB Medina-Ruiz S Mitros T , et al. Genome and transcriptome mechanisms driving cephalopod evolution. Nat Commun 2022; 13: 2427. 35508532 10.1038/s41467-022-29748-w PMC9068888

[7] Ponte G Andrews P Galligioni V , et al. Cephalopod welfare, biological and regulatory aspects: an EU experience. In: Carere C Mather J (eds) The welfare of invertebrate animals. Cham: Springer, 2019, pp.209–228.

[8] Birch J Burn C Schnell A , et al. Review of the evidence of sentience in cephalopod molluscs and decapod crustaceans. London: London School of Economics and Political Science, 2021.

[9] European Parliament and Council of the European Union. Directive 2010/63/EU of the European Parliament and of the Council of 22 September 2010 on the protection of animals used for scientific purposes, https://eur-lex.europa.eu/legal-content/EN/ALL/?uri=CELEX:32010L0063 (2010, accessed April 2023).

[10] Vidal EAG Villanueva R Andrade JP , et al. Cephalopod culture: current status of main biological models and research priorities. In: Vidal EAG (ed) Advances in marine biology. London, UK: Academic Press, 2014, pp.1–98. 10.1016/B978-0-12-800287-2.00001-9 24880794

[11] Villanueva R Sykes AV Vidal EAG , et al. Current status and future challenges in cephalopod culture. In: Iglesias J Fuentes L Villanueva R (eds) Cephalopod culture. Dordrecht: Springer Netherlands, 2014, pp.479–489.

[12] Capaz JC Hernández-Brooke D Balvet S , et al. Control of zootechnology leads to improved cuttlefish (*Sepia officinalis*, L.) reproduction performance up to pre-industrial levels. Front Marine Sci 2020; 7: 112.

[bibr13-00236772231176347] Hanlon RT Claes MF Ashcraft SE , et al. Laboratory culture of the sepiolid squid *Euprymna scolopes*: a model system for bacteria–animal symbiosis. Biol Bull 1997; 192: 364–374. 28581841 10.2307/1542746

[14] Iglesias J Fuentes L Villanueva R (eds). Cephalopod culture. Dordrecht: Springer Netherlands, 2014.

[15] Jacquet J Franks B Godfrey-Smith P , et al. The case against octopus farming. Issues Sci Technol 2019; 35: 37–44.

[16] Lara E. Welfare and environmental challenges of octopus aquaculture. Aquatic Animal Welfare Conference 2020, YouTube, 2020. https://youtu.be/wS5r0DJgHWA (2020, accessed April 2023)

[17] Pieroni EM Sykes A Galligioni V , et al. Review on the methods of capture and transport of cephalopods for scientific purposes, https://felasa.eu/Announcements/id/38; doi.org/10.53124/cephres.202201 (2022, accessed September 2022).

[18] Fiorito G Affuso A Basil J , et al. Guidelines for the care and welfare of cephalopods in research – a consensus based on an initiative by CephRes, FELASA and the Boyd Group. Lab Anim 2015; 49: 1–90. 10.1177/0023677215580006 26354955

[19] Commission of the European Communities. Commission Recommendation of 18 June 2007 on guidelines for the accommodation and care of animals used for experimental and other scientific purposes (notified under document number C(2007) 2525), https://eur-lex.europa.eu/legal-content/EN/TXT/?uri=CELEX%3A32007H0526 (2007, accessed April 2023).

[20] Globefish. World Congress on cephalopods: markets and trade, http://www.fao.org/in-action/globefish/news-events/details-news/en/c/449821/ (2016, accessed January 2021).

[21] Santarelli M. La pesca del polpo (*Octopus vulgaris*) nel golfo di Napoli. Bull Inst Océanogr 1932; 597: 1–6.

[22] Rathjen WF. Cephalopod capture methods: an overview. Bull Marine Sci 1991; 49: 494–505.

[23] Carlson BA. Collection and aquarium maintenance of Nautilus. In: Landman NH Saunders WB (eds) Nautilus: the biology and paleobiology of a living fossil. Dordrecht: Springer, 2010, 1991, pp.563–578.

[24] Watanuki N Kawamura G. A review of cuttlefish basket trap fishery. South Pacific Stud 1999; 19: 31–48.

[25] Montgomery MK McFall-Ngai M. Embryonic development of the light organ of the sepiolid squid *Euprymna scolopes* Berry. Biol Bull 1993; 184: 296–308. 29300543 10.2307/1542448

[26] Matsumoto G. Transportation and maintenance of adult squid (*Doryteuthis bleekeri*) for physiological studies. Biol Bull 1976; 150: 279–285. 1268299 10.2307/1540474

[27] Chabala LD Morello RS Busath D , et al. Capture, transport, and maintenance of live squid (*Loligo pealei*) for electrophysiological studies. Pflugers Arch 1986; 407: 105–108. 3737375 10.1007/BF00580729

[28] Lane FW. Kingdom of the octopus; the life history of the Cephalopoda. New York: Sheridan House, 1960.

[29] Carreira GP Gonçalves JM. Catching *Octopus vulgaris* with traps in the Azores: first trials employing Japanese baited pots in the Atlantic. Mar Biodivers Rec 2009; 2: e114.

[30] Sauer WHH Gleadall IG Downey-Breedt N , et al. World octopus fisheries. Rev Fish Sci Aquac 2021; 29: 279–429.

[31] Berka R. The transport of live fish: a review. Rome: European Inland Fisheries Advisory Commission (EIFAC); FAO – Food and Agriculture Organization of the United Nations, 1986.

[32] Grimpe G. Pflege, Behandlung und Zucht der Cephalopoden fur zoologische und physiologische Zwecke. In: Äberhalden E (ed) Handbuch der biologischen Arbeitsmethoden. Berlin: Verlag Urban & Schwarzenberg, 1928, pp.331–402.

[33] De Sio F Hanke FD Warnke K , et al. E pluribus octo – building consensus on standards of care and experimentation in cephalopod research; a historical outlook. Front Physiol 2020; 11: 645. 32655409 10.3389/fphys.2020.00645 PMC7325997

[34] Boyle PR. Ventilation rate and arousal in the octopus. J Exp Mar Biol Ecol 1983; 69: 129–136.

[bibr35-00236772231176347] Katsanevakis S Stephanopoulou S Miliou H , et al. Oxygen consumption and ammonia excretion of *Octopus vulgaris* (Cephalopoda) in relation to body mass and temperature. Mar Biol 2005; 146: 725–732.

[bibr36-00236772231176347] Melzner F Mark FC Pörtner HO. Role of blood-oxygen transport in thermal tolerance of the cuttlefish, *Sepia officinalis*. Integr Comp Biol 2007; 47: 645–655. 21672869 10.1093/icb/icm074

[37] Boucher‐Rodoni R Mangold K. Ammonia production in cephalopods, physiological and evolutionary aspects. Mar Fresh Behav Physiol 1995; 25: 53–60.

[38] Lekang OI. Transport of live fish. Aquaculture engineering. Chichester, UK*:* John Wiley, 2013, pp.328–338.

[39] Gleadall IG. Low dosage of magnesium sulphate as a long-term sedative during transport of firefly squid, *Watasenia scintillans*. J Exp Mar Biol Ecol 2013; 447: 138–139.

[40] Hanlon RT. Maintenance, rearing, and culture of teuthoid and sepioid squids. In: Gilbert DL Adelman WJ Arnold JM (eds) Squid as experimental animals. Boston, MA: Springer, 1990, pp.35–62.

[41] Villanueva R Vidal EAG Fernández-Álvarez FÁ , et al. Early mode of life and hatchling size in cephalopod molluscs: influence on the species distributional ranges. PLoS One 2016; 11: e0165334. 27829039 10.1371/journal.pone.0165334 PMC5102429

[42] Deryckere A Styfhals R Vidal EAG , et al. A practical staging atlas to study embryonic development of *Octopus vulgaris* under controlled laboratory conditions. BMC Dev Biol 2020; 20: 7. 32299349 10.1186/s12861-020-00212-6 PMC7164171

[43] Boletzky S. Recent studies on spawning, embryonic development, and hatching in the Cephalopoda. Adv Mar Biol 1989; 25: 85–115.

[44] Seibel BA Robison BH Haddock SHD. Post-spawning egg care by a squid. Nature 2005; 438: 929–929. 16355206 10.1038/438929a

[45] Bush SL Hoving HJT Huffard CL , et al. Brooding and sperm storage by the deep-sea squid *Bathyteuthis berryi* (Cephalopoda: Decapodiformes). J Mar Biol Assoc UK 2012; 92: 1629.

[46] Rosa R Pimentel MS Boavida-Portugal J , et al. Ocean warming enhances malformations, premature hatching, metabolic suppression and oxidative stress in the early life stages of a keystone squid. PLoS One 2012; 7: e38282. 22701620 10.1371/journal.pone.0038282 PMC3368925

[bibr47-00236772231176347] Boletzky Sv. Biology of early life stages in cephalopod molluscs. Adv Mar Biol 2003; 44: 143–203. 12846042 10.1016/s0065-2881(03)44003-0

[bibr48-00236772231176347] Nabhitabhata J Asawangkune P Amornjaruchit S , et al. Tolerance of eggs and hatchlings of neritic cephalopods to salinity changes. Phuket Marine Biological Center Special Publication 2001; 25: 91–99.

[49] Bower JR Sakurai Y. Laboratory observations on *Todarodes pacificus* (Cephalopoda: Ommastrephidae) egg masses. Am Malacol Bull 1996; 13: 65–71.

[50] Spreitzenbarth S Jeffs A. Egg survival and morphometric development of a merobenthic octopus, *Octopus tetricus*, embryos in an artificial octopus egg rearing system. Aquaculture 2020; 526: 735389.

[51] Iglesias J Sánchez FJ Bersano JGF , et al. Rearing of *Octopus vulgaris* paralarvae: present status, bottlenecks and trends. Aquaculture 2007; 266: 1–15.

[52] Araújo J Matias AC Pousão‐Ferreira P , et al. Development of a method for live octopus (*Octopus vulgaris*) transportation for long distance at high densities. Aquac Res 2020; 51: 3751–3759.

[53] Barragán-Méndez C Sobrino I Marín-Rincón A , et al. Acute-stress biomarkers in three octopodidae species after bottom trawling. Front Physiol 2019; 10: 784. 31293450 10.3389/fphys.2019.00784 PMC6603232

[54] Council of the European Union. Council Regulation (EC) No 1/2005 of 22 December 2004 on the protection of animals during transport and related operations and amending Directives 64/432/EEC and 93/119/EC and Regulation (EC) No 1255/97, https://eur-lex.europa.eu/legal-content/en/ALL/?uri=CELEX%3A32005R0001 (2004, accessed April 2023).

[55] Smith AJ Clutton RE Lilley E , et al. PREPARE: guidelines for planning animal research and testing. Lab Anim 2018; 52: 135–141. 28771074 10.1177/0023677217724823 PMC5862319

[56] Smith AJ Clutton RE Lilley E , et al. Improving animal research: PREPARE before you ARRIVE. BMJ 2018; 360: k760. 29472181 10.1136/bmj.k760

[57] Kilkenny C Browne WJ Cuthill IC , et al. Improving bioscience research reporting: the ARRIVE guidelines for reporting animal research. PLoS Biol 2010; 8: e1000412. 20613859 10.1371/journal.pbio.1000412 PMC2893951

[58] Percie du Sert N Ahluwalia A Alam S , et al. Reporting animal research: explanation and elaboration for the ARRIVE guidelines 2.0. PLoS Biol 2020; 18: e3000411. 32663221 10.1371/journal.pbio.3000411 PMC7360025

[59] Jones JB. Environmental impact of trawling on the seabed: a review. N Z J Mar Freshw Res 1992; 26: 59–67.

[bibr60-00236772231176347] Gray JS Dayton P Thrush S , et al. On effects of trawling, benthos and sampling design. Mar Pollut Bull 2006; 52: 840–843. 16945683 10.1016/j.marpolbul.2006.07.003

[bibr61-00236772231176347] Hiddink JG Jennings S Sciberras M , et al. Global analysis of depletion and recovery of seabed biota after bottom trawling disturbance. Proc Natl Acad Sci U S A 2017; 114: 8301–8306. 28716926 10.1073/pnas.1618858114 PMC5547586

[62] Johnson AF Gorelli G Jenkins SR , et al. Effects of bottom trawling on fish foraging and feeding. Proc R Soc B Biol Sci 2015; 282: 20142336. 10.1098/rspb.2014.2336 PMC4286059 25621336

