# Supplementary material for: FELASA Working Group report: Capture and transport of live cephalopods – recommendations for scientific purposes
Source: Lab Anim. 2023 Nov 3;58(2):170–82. doi: 10.1177/00236772231176347 (PMC11102646; doi:10.1177/00236772231176347)
Supplement: sj-pdf-1-lan-10.1177_00236772231176347 - Supplemental material for FELASA Working Group report: Capture and transport of live cephalopods – recommendations for scientific purposes [file sj-pdf-1-lan-10.1177_00236772231176347.pdf]

Supplementary Information to

FELASA Working Group Report

Capture and Transport of live cephalopods: recommendations for scientific purposes

A.V. Sykes (Convenor)[1,a], V. Galligioni[2,b], J. Estefanell[3,c], S. Hetherington[4,d], M. Brocca[5],

J. Correia[6], A. Ferreira[7], E.M. Pieroni[8,e], G. Fiorito[8,9,*]

[1] CCMAR – Centro de Ciências do Mar do Algarve, Universidade do Algarve, Campus de Gambelas, 8005-139 Faro, Portugal

[2] Comparative Medicine Unit, Trinity College Dublin, Ireland

[3] Ciclo Superior Cultivos Acuicolas, Instituto de Educacion Secundaria les Profesor Cabrera Pérez, Las Palmas, Spain

[4] CEFAS - Centre for Environment, Fisheries and Aquaculture Science

[5] TECNIPLAST S.p.A., via I Maggio, 6, 21020 Buguggiate (VA), Italy

[6] Flying Sharks, Rua do Farrobim do Sul 116, 9900-361 Horta – Portugal

[7] Praceta do sol lote 4 nº57 3ºD, 2775-795 Lisboa, Portugal

[8] Association for Cephalopod Research 'CephRes', Italy

[9] Department of Biology and Evolution of Marine Organisms, Stazione Zoologica Anton Dohrn, Villa Comunale, Napoli, Italy

*Corresponding author: Graziano Fiorito
email: graziano.fiorito@szn.it; rd_innovation@cephalopodresearch.org

Representing FELASA Members:

[a] SPCAL – Sociedade Portuguesa de Ciências em Animais de Laboratório, Portugal

[b] AISAL - Associazione Italiana per le Scienze degli Animali da Laboratorio, Italy

[c] SECAL - Sociedad Española para las Ciencias del Animal de Laboratorio, Spain

[d] LASA - Laboratory Animal Science Association, UK

[e] Corresponding Member, Association for Cephalopod Research 'CephRes' a non-profit organization, Italy

Keywords: Capture, transport, cephalopods, Directive 2010/63/EU, welfare, training

Table of Contents

Suppl_RN: Reference to the Ancillary work and other Notes ... 3
Other Notes about Cephalopods and their Biology ... 3
Suppl_1: Legislative framework ... 4
Suppl_1.1: Competence and attitude of the personnel involved in capture and transportation ... 7
Suppl_1.2: Principles for Transport, documentation and planning of the journey ... 7
Suppl_2: Different forms, different methods: considerations for Capture and Transport of different cephalopods' life stages ... 11
Suppl_3: Other Recommendations for Transport of live cephalopods ... 12
Suppl_3.1: General Requirements ... 14
Suppl_4: Supporting info to the overview of Capture and Transport Methods ... 14
4.1: *Nautilus* ... 15
4.2: Cuttlefish ... 16
4.3: Sepiolids ... 17
4.4: Squid ... 18
4.5: Octopus ... 19
Suppl_5: Topics and Learning outcomes of an accredited course for collectors and transporters of live wild cephalopods to be used in scientific research ... 21
General principles ... 21
Suppl_Box 1: Training Plan ... 23
References ... 26

Suppl_RN: Reference to the Ancillary work and other Notes

Ancillary work to Sykes et al. (2023):

Eleonora Maria PIERONI, Antonio V. SYKES (Convenor), Viola GALLIGIONI, Juan ESTEFANELL, Stuart HETHERINGTON, Marco BROCCA, Joao CORREIA, André FERREIRA, Graziano FIORITO (2022). Review on the methods of capture and transport of cephalopods for scientific purposes – Outcomes of FELASA Working Group 'Capture and Transport of Cephalopods. *CephRes Publications and Reference Docs* [Online]. FELASA & Association for Cephalopod Research CephRes-ETS, doi: 10.53124/cephres.202201 [accessed September 2022][1].

Other Notes about Cephalopods and their Biology

As mentioned in the main text, cephalopod molluscs are animals of great interest for commercial purposes being recognized for the great value as food for human consumption.[2, 3] These animals account for a 5% of the marine capture volume worldwide[4, 5], with a significative increase in their demand, though recently slowed down by COVID-19 outbreak summarized in various instances, as follows:

i. Cephalopods sector suffering under COVID-19, http://www.fao.org/in-action/globefish/market-reports/resource-detail/en/c/1263830/
ii. Cephalopods sector hit hard by COVID-19, http://www.fao.org/in-action/globefish/market-reports/resource-detail/en/c/1306820/
iii. Industry still struggling, but hopes for improvement in 2021, http://www.fao.org/in-action/globefish/market-reports/resource-detail/en/c/1253479/
iv. Varied supply situation, higher prices, https://www.fao.org/in-action/globefish/market-reports/resource-detail/en/c/1605547/

Suppl_1: Legislative framework

We analysed the available legislation and recommendations regarding capture and transport of aquatic animals, and identified more than 20 different documents. Table S1 lists the sources we considered. Here we do not provide a detailed overview about the legislative framework, but suggest the Reader to refer to the ancillary work[1] for completeness and to table 1 therein.

Table S1. List of documents considered for the analysis of the legislative framework relevant to the capture and transport of aquatic animals. Dates of documents are referred to their entry into force, in most of the cases; for Packer's Guidelines we refer to CITES the Conference of the Parties held at San José, Costa Rica. For details see the ancillary work (Pieroni et al.[1]).

Year	Document	Reference
1979	Packer's Guidelines Inv1/Aquatic invertebrates	https://cites.org/eng/resources/transport/inv1.shtml
1986	86/609/EEC	https://eur-lex.europa.eu/legal-content/EN/ALL/?uri=celex%3A31986L0609
1991	91/67/EEC	https://eur-lex.europa.eu/legal-content/en/ALL/?uri=CELEX%3A31991L0067
1993	Olfert et al. (1993)[6]	https://awionline.org/lab-animal-search/canadian-council-animal-care-olfert-e-d-cross-b-m-et-al-1993-guide-care-and-use
2005	EC No 1/2005	https://eur-lex.europa.eu/legal-content/EN/ALL/?uri=celex%3A32005R0001
2005	Guidelines for the Transport of Laboratory Animals	https://journals.sagepub.com/doi/pdf/10.1258/0023677052886493
2006	ETS No.193	https://www.coe.int/en/web/conventions/full-list?module=treaty-detail&treatynum=193
2007	2006 No. 3260	https://www.legislation.gov.uk/uksi/2006/3260/pdfs/uksi_20063260_en.pdf
2007	2007/526/EC	https://eur-lex.europa.eu/legal-content/EN/TXT/?uri=celex%3A32007H0526
2010	EAZA Position Statement on Council Regulation 1/2005: Protection of Animals during Transport	https://www.eaza.net/assets/Uploads/Position-statements/2010-12-EAZA-Position-Paper-on-Animal-Transport-final.pdf
2011	Guide for the Care and Use of Laboratory Animals	https://grants.nih.gov/grants/olaw/guide-for-the-care-and-use-of-laboratory-animals.pdf
2013	Australian code for the care and use of animals for scientific purposes 8th Edition 2013	https://www.nhmrc.gov.au/about-us/publications/australian-code-

Year	Document	Reference
		care-and-use-animals-scientific-purposes
2013	Directive 2010/63/EU[7]	https://eur-lex.europa.eu/LexUriServ/LexUriServ.do?uri=OJ:L:2010:276:0033:0079:en:PDF
2013	Conf. 10.21 (Rev. CoP16)	https://cites.org/sites/default/files/document/E-Res-10-21-R16.pdf
2014	NC3Rs' best practice for animal transport	https://nc3rs.org.uk/3rs-resources/best-practice-animal-transport
2022	Aquatic Animal Health Code	https://www.woah.org/en/what-we-do/standards/codes-and-manuals/aquatic-code-online-access

Hereunder, we only briefly overview the main aspects relevant to the legislative framework encompassing aquatic species' wellbeing in the context of commercial and trade purposes.

The Council Directive 91/67/EEC and the following European legislations regulating the transport of animals for commercial or experimental purposes, is centred on vertebrate species, mainly terrestrial animals.

Interestingly, the United Kingdom legislation extended the concept of animal to which the EC No 1/2005 should be referred including all the "cold-blooded invertebrates".[8]

Furthermore, and as relevant to transport of wildlife, both the Office International des Epizooties (OIE) and CITES[9] consider all animals listed in the Live Animal Regulation (LAR), including cephalopods (for review see Pieroni et al.[1]).

CITES has also published the "Packer's guidelines" (https://cites.org/eng/resources/transport/inv1.shtml) for aquatic invertebrates, and as we assume cephalopods are included therein. In these guidelines detailed instructions about general welfare, the arrangements of transport and shipment as well as the design of the container are provided; no species-specific information is included. Guidelines concerning the capture and transport of laboratory animals (mainly vertebrates) have been published by the US Institute

for Laboratory Animal Research (ILAR), these include a mention to cephalopods (see section 'Applicability and Goals'[10]; see also Pieroni et al.[1] for review).

In addition, it is since 1993 that the Canadian Council of Animal Care refers in its welfare Act to any non-human vertebrates and cephalopods.[6] The document includes extensive guidelines on procurement and transportation of purpose-bred animals and wildlife, however without specific mention to cephalopod molluscs (see Pieroni et al.[1] for review). The Australian legislation for the use of animals for laboratory purposes includes cephalopods and obliges to minimise the risk of injury or stress-induced diseases during their capture and transport. In the Australian law, capture and handling of wildlife (cephalopods included) must comprise: «***i.*** the involvement of a sufficient number of competent people to restrain animals in a quiet environment and prevent injury to animals and handlers; ***ii.*** chemical restraint (e.g., sedatives) where appropriate, if the period of handling is likely to cause harm, including pain and distress to animals; ***iii.*** restraint and handling of animals for the minimum time needed to achieve the aims of the project or activity; ***iv.*** making provisions for captured animals that are ill or injured, including treatment of pain and distress».[11] Again, no species-specific details for cephalopods are provided.

As reviewed in Pieroni et al. (2022)[1] the available legislation and guidelines also include information about proper ***i.*** documentation and planning of the journey, ***ii.*** methods and means of transport including design of containers, ***iii.*** health assessment, food, and water supplies for the animals, ***iv.*** the need of acclimatization before and after transport of wild animals.

In sum, all the documents we considered (see Table S1 and Pieroni et al., 2022[1]) have very little information (or any) relevant to cephalopod molluscs.

Suppl_1.1: Competence and attitude of the personnel involved in capture and transportation

The team of collectors or fishers and shippers has to be properly trained for the scope, and it is responsibility of Authorities to verify that this will occur. Depending on the cases, the team should avail of the support and advice of a Designated Veterinarian who shall check the health status of the animals and shall take the proper actions to avoid or end suffering.

Annex IV of the EU Council Regulation No 1/2005[12] states that drivers and attendants [as referred to in Article 6(5) and Article 17(1)] shall have successfully completed the training and have passed an examination, ensuring that examiners are independent. The courses should include technical and administrative aspects of Community legislation concerning the protection of animals during transport, including animal physiology (e.g., drinking and feeding needs), animal behaviour and the concept of stress. Moreover, practical aspects of animal handling and of the emergency care both for the personnel and the animals should be considered in the training program assuring that personnel involved (drivers) should know how the driving behaviour might impact on the welfare of the transported animals thus also impacting the quality of meat, if the animals are destined for food consumption (see Annex IV)[12].

Suppl_1.2: Principles for Transport, documentation and planning of the journey

Regardless of the purpose of capture and transport of animals, the main principles included in the guidelines and legislations (for review see Pieroni et al.[1]) concerning the planning phases prior collection and transport, may be applied to cephalopods after the due considerations.

Transport, and in particular long journeys, might be detrimental for the health conditions of animals[12, 13] thus, all the planning should be done in advance and the transport should be carried out without delay to the place of destination.

According to the available knowledge that with increased duration of confinement for transport, both consumption of available oxygen and detrimental changes in water chemistry (e.g., accumulation of ammonia, carbon dioxide and depletion of oxygen), we can distinguish (review in Fiorito et al.[14]):

i. **Short duration journey** (< 2h; e.g., from local capturing site to local establishment). Although this is most likely the less stressful situation, there are important conditions that should be monitored before and during the trip to the final location (e.g., pH, temperature and salinity, saturation of oxygen, light exposure). Vibration, noise, and any other kind of interference should be limited and/or avoided; attention to the infliction of any physical damage should be constantly given.

ii. **Long duration journey** (> 2h; e.g., between towns, countries and/or intercontinental). The same precautions described for the short duration trip applies also to this situation. However, considering that the journey might imply the shipping of the animals by sea or by air, special attention should be given to the containers used for this kind of transportations. Care should be provided to include the documentation concerning management of the welfare state of the animals during and after the journey.

During a short-duration transport, plastic bags may be replaced with large plastic buckets or boxes with a lid containing sufficient pre-oxygenated seawater to allow the animal to be completely immersed.[14-18] For long-duration transport, - and similarly to fish[19] - considering animals' body size, the cephalopod should be placed with adequate volume of seawater and oxygen-enriched air in double common aquarium aerated bags (see also Table 2). For transport periods over 12 hours, aeration and oxygenation may be necessary being careful not to induce distress to the animals; water turbulence and bubbling can cause air entrapment in the mantle cavity or produce microbubbles affecting the integrity of the mucus layer on the skin. Although a transparent bag is the better choice, because it allows easy inspection, arranging things in order to have the external, second bag dark in colour (dark blue or green) is considered here the best option, since it offers shading to the animals and not bright environmental background. The alternative should be to have the internal side of the container box, dark in colour. Sealed holding bags containing oxygenated seawater should be placed into

insulated boxes (e.g., Styrofoam) to ensure that a temperature, appropriate to the species, is maintained during transport[14]. Bags should be packed with cushioning material (e.g., paper, Styrofoam pellets) to ensure they do not move during transport and the external shipping box should report the labels: "this side up" and "live animals" (see below: Suppl_3: Other Recommendations for Transport of live cephalopods, and Suppl_3.1: General Requirements).

In Europe, but also according to other national legislations (see table 1 in Pieroni et al.[1]), every transportation should be preceded by accurate planning of the journey and shall be accompanied by a proper documentation stating: **a)** the origin and their ownership; **b)** the place of departure; **c)** the date and time of departure; **d)** the intended place of destination; **e)** the expected duration of the intended journey (see Chapter II, Art. 4 and Appendix included in EU Council Regulation No 1/2005).[12] The regulation recommend that a competent scientific committee shall be consulted about the duration and the route plans of animal transport, and according to a harmonized European model, certificate for transporters must be presented. Authorities shall take the necessary measures to prevent or reduce to a minimum any delay during transport (e.g., programming special arrangements at the place of transfers, exit points and border inspections to give priority to the transport of animals) but resting periods at specific control posts shall be planned if the journey is longer [Art. 22(1)].[12] Furthermore, the consignment shall be done immediately unless the detainment is necessary for the health of the animal or for public safety, such as possible spread of zoonosis if there are some diseased animals [Art. 22(2)].[12] For such a reason, veterinary checks at borders' inspection include the analysis of the welfare conditions in which the animals are transported. Depending on the arrangements, veterinary documents likely accompany animals from origin.

Further documentation is needed if transporting wild, timid or dangerous species, providing instructions about feeding, watering and any special care

required for them (see Chapter II, Section 1.3 of the EU Council Regulation No 1/2005).[12]

The person planning the journey has to bring and compile a journey log, which reports in detail any daily event including animals' health status, any intervention performed and any detour from the original plan (see Annex II)[12]. As for the regulation of the international transport of animals the European Convention for the Protection of Animals during International Transport (ETS No.193[20]) applies.

On the basis of EU legislation and other documents - such as ETS No.193 and Council Recommendation 2007/526/EC (see Pieroni et al.[1]) - specifically addressed to experimental animals, LASA produced a WG Report 'Guidance on the transport of laboratory animals'[21] which lists all the documentation needed during the planning of the journey (see Paragraph 3.3 of the WG Report[21]). Of course, the number and type of documents to be filled up depend on the journey type, species, microbiological status, and route.

However, the following information should not be missing (see also the ancillary work for details[1]):

a. ***shipment documentation details*** such as waybill number or IATA Shipper's certificate (for Air transport), import licenses issued by the State Veterinary Service, CITES permits where necessary (for intra-European and Third-country shipping), invoices for Customs purposes, health certificate of the animal transported signed by the Designated Veterinarian, journey log or transfer authorizations from specific bodies that regulate laboratory animals' use

b. ***animal details*** such as species, strain, scientific name, number, sex, age, weight, identification numbers or any special requirements resulting from phenotype

c. ***personnel details*** such as contact information of sender, intermediaries, consignee, shipper/carrier, veterinarian

d. ***crates*** with date and times the animals were packed loaded, and departed with clear 'Live animals' and orientation arrows labels; ***v.*** expected events, such as proposed and actual rest periods, pre-journey

review of plan by consignor and post-journey review of plan by new owner.[21]

Suppl_2: Different forms, different methods: considerations for Capture and Transport of different cephalopods' life stages

It is essential to take into account the life stage of the target cephalopod species.
As discussed in the main text, collection of eggs and standardized transport of them have been proposed as an alternative to the transfer of post-natal, juveniles and adults, whenever possible. This of course depends on species and in some cases considered easier to manage.
The Directive 2010/63/EU considers the protection of this taxon from hatching, thus eggs should be excluded formally.
However, the capture and transport of eggs do not circumvent the need of establishing recommendations and standardized protocols for capture and transport of cephalopods for research purposes. In analogy to what required for post-natal forms, accurate, reliable and standardized protocols should be attentively developed in the case eggs will be collected from the wild and transported to their destination for research purposes.
Another aspect to consider is whether maternal care is required because of cephalopod species biology (e.g., the case of incirrate octopods or oceanic squids[22-27]). Maternal care is seen as a critical factor for the proper embryonic development, to the best of our knowledge and only a few attempts to substitute the natural setting provided significant results.[28, 29]

Cephalopod hatchlings are either miniature adults or planktonic paralarvae with relatively short arms and limited swimming ability[30-32] and represent extremely

delicate developing forms, very sensitive to any insult or change in the water parameters from the site of collection to the containers. Nevertheless, the majority of studies (for review see table 2 in Pieroni et al.[1]) used trawls and bongo nets for collection, with few reporting also that animals which resulted damaged were excluded from the experiments (for example see Otero et al.[33]). A few notes about capture and transport of paralarval stages of cephalopods is given in the ancillary work to this paper.[1]

The collection of juveniles is a challenging task because these may be selective and with rigid feeding requirements;[34] appropriate cautions should be applied in these circumstances, because a traumatic capture might affect highly feeding and behavioural responses that may translate into fatal conditions.

Adults have also important temperature, salinity and pH requirements as most of the cephalopods are stenotherm and stenohaline. It follows that the inappropriate capture and transport of any individual at any life stage from the wild might result in high mortality, and therefore considerable species-specific care is needed if viable animals have to be returned to the laboratory.[16, 34-36] Further information and summary of requirements are provided in the ancillary review.[1]

Suppl_3: Other Recommendations for Transport of live cephalopods

Prior to transport, and before long-journeys, wild-caught cephalopods should be acclimated to captivity in tanks by gradually moving the animals in the new seawater. Depending on the new water "composition" (e.g., in terms of salinity, pH, temperature) acclimatisation can be different in time. Health and welfare

should be monitored during this phase, checking for good appetite, presence of any skin lesions and potential unusual behaviour.

If the journey to the lab is brief, small sepioids and octopuses could be temporarily placed in containers with seawater. If temperature, pH and oxygen content values change, renewal of seawater is mandatory.[15, 16] For transport lasting more than one or two hours, small numbers of small-sized individuals could be carried in cooled boxes where bags are positioned. Bags should contain a single individual with sufficient seawater to cover the animals (i.e. a polythene bag about 1/3 filled with seawater with oxygen filling the remaining space). Survival for 8-10 h was reported to be easily possible by sealing and keeping the bags with temperature that is kept almost constant.

Another factor to consider is food deprivation. It is suggested to prevent animals from feeding for 24 hours before long-term journeys because it helps keeping metabolic rate under baseline, possibly limiting ammonia build-up during transport. However, food deprivation depends on the species, animal normal feeding frequency, oro-anal transit time and renal ammonium ion excretion for the species.[37]

Sedation is not essential and is not recommended for transport of most cephalopods. However, sedation methods have been utilized for the transport of some species and with controversial results in various circumstances. Interestingly Grimpe[16] suggested that very long duration transportation - i.e. requiring more than two days - should be achieved in steps allowing ‘resting’ periods in appropriate locations which is what nowadays is indicated by the European and international legislations for the transport of live animals (for details see Pieroni et al.[1]).

Suppl_3.1: General Requirements

As anticipated above, each trip/journey/shipment must be preceded by the presentation of the suitable documentation and planning according to the national or international legislation. Depending on the mean of transport utilized, the correct adaptation should be followed to ensure the welfare of the animals being transported. The holding containers in which animals are stored should be composed of 1/3 seawater (preferably at the seawater temperature at the collection site), and 2/3 oxygen which should not be pumped in to avoid/prevent the generation of bubbling in the mantle of the animals. Water collection on site should be promoted. During the transfer it is mandatory to avoid air exposure and dehydration, together with sudden temperature changes from the sea to the tank.

As reiterated in many instances, animals should be food-deprived prior and during the trip/journey/shipment (depending on the duration and the species), in order to prevent ammonia waste upbuilding in the seawater. For longer journey, water should be completely changed after 24 hours with water taken from the capture site which should be carried along and properly monitored. Other seawater with the same composition can also be used, but it might cause distress for the animals; therefore, accurate monitoring of animals during the transfer/change of seawater should be considered.

Vibration, noise and direct light must be kept to a minimum to minimize further stress and harm.

The welfare state of the animals should be periodically checked during the journey or at the resting place (if the journey is very long) and expert (or trained) personnel should be able to take the most humane decision in case of harmed specimen.

Suppl_4: Supporting info to the overview of Capture and Transport Methods

The following text provides complementary and explanatory information in support to recommendations summarized in Tables 1 and 2 (main text). For each

topic, we refer to abbreviations and/or acronyms utilized in the Table(s), species/taxa (when taxon-specific the arrangement of the text follows using the taxon as header), capture methods (whenever necessary), supporting information and references, as source of information.

W, E: Welfare & Environmental issues – The method of capture and/or transport is considered to raise welfare issues, i.e. to the possibility of inducing PSDLH in cephalopods. For environmental 'issues', we (as also stated in many studies; for review see[1, 38]) consider that **Trawl** might cause environmental damage due to the alteration and/or destruction of sea floors.[39-44] In addition, the method is considered not-selective which results in by catch.[45]

It is noteworthy to mention that, based on the current status of the species - over 55% of them considered as Data deficient by the IUCN (https://www.iucnredlist.org/search?query=cephalopods&searchType=species; last visited April 2023), there are potential additional risks to face when the most recommendable method is not possible to apply in capturing the species, and/or a less recommendable method is the only possible. Additional risks for conservation of the target species (i.e. cephalopods) are linked to those related to other taxa living in the same fishing area, and - as shown by various authors – our knowledge of the potential impact is only limited. As for cephalopods, possible pilot studies to address these issues and cross-analysis with historical data in given geographical areas should be considered in the near future.

GPS: GPS or monitoring systems may be applied to the gear, thus allowing monitoring of the exact location where the capture occurred.

4.1: *Nautilus*

The most recommended capture methods are Baited[46-51] or Light Traps[52] (**BLT**) which are suitable for both juvenile (**Juv**) and adults (**Ad**). Trawl is not

recommended because of welfare and environmental issues (W, E)[34, 45] and other capture methods are not generally adopted.

For transport, the best method is **Box** in which few specimens can be stored together when this is unavoidable. In the Table 2 we refer to: **M** (no more than 4 animals in a 20 L box, suitable for both small and large specimen), in chilled water (**ChW**, 15-18°C); tightly sealed and contained in a larger Styrofoam box; reported satisfactory survival (**S**) for travel up to 4-24 h[47]. Tanks can be used as containers for holding bags (**Chb**), should be transparent to facilitate inspection if required. Ensure that an appropriate temperature is maintained during transport.

Plastic bags are not recommended because these animals tend to bite them.[47]

4.2: Cuttlefish

The most recommended capture methods are Basket Traps[53-55] (**BkT**) or Cuttlefish Traps[56] (**CtT**), larger and lighter than the ones utilized for squids. They are size-selective (**SzS**) and employed for catching adults (**Ad**). Funnel entrance is in the smaller side and opening "door" on the top to remove the catches.[57]

Seabed is often included as substrate (**Sub**). Females and potentially attached eggs should not be taken for conservation issues unless properly justified. The justification provided for the use in scientific research should warrant adequate consideration about conservation issues.

Trammel nets (**TN**) can also be employed. These are large enough to catch a reasonable number of animals without excessive constraint[58] and are suitable for both juveniles (**Juv**) and adults (**Ad**).

Pots are not generally adopted while Trawl and Jigs are not recommended for welfare and environmental issues (W, E).[34, 45]

For transport, the most suitable method are plastic bags which should be filled with seawater and oxygen, and properly sealed (e.g., twisted at the top and folded over) and doubly secured.[14, 19] Few animals per bag can be placed

depending on the volume of the bag, the duration of the journey and on the size of the animals (a maximum of 20 cuttlefishes of 30-40 DML were maintained together in 6L of seawater[38]). However, our recommendation is to ship cuttlefish in individual bags. Animals could survive up to 12h.

Box and tanks can be used as well, as containers for holding bags (**Chb**) for both juveniles (**Juv**) and adults (**Ad**) to ensure that an appropriate temperature is maintained during transport. Substrate (**Sub**) can be added if the journey is short as the addition of seaweed or other organic material can be subjected to decomposition processes thus reducing water quality and oxygen, compromising animal welfare during longer journey; sand poses the risk of H_2S release from fouled sediments.

4.3: Sepiolids

The most recommended capture methods are Dipnets (**DN**) or Seine nets (**SN**) which are large enough to catch a reasonable number of animals without excessive constraint.[59-62] These are suitable for both juveniles (**Juv)** and adults (**Ad)**. Jigs are not generally adopted while traps and pots are not recommended, because these specimens are too small for this capture method (size issues, **SzI**). Trawl is not recommended[34, 45] for welfare and environmental issues (**W**, **E**).

For transport the most suitable method are plastic bags which should be filled with seawater and oxygen, and properly sealed (e.g., twisted at the top and folded over) and doubly secured.

More than one animal per bag can be placed because of the small size of these species, depending on the volume of the seawater and the duration of the journey. Reported survival is of up to 21h.[63]

Box and tanks can be used as well, containers for holding bags (**Chb**) for both juveniles (**Juv**) and adults (**Ad**); this also helps to ensure that an appropriate temperature is maintained during transport. The containers can be provided with

substrate (**Sub**) if the journey is short as the addition of seaweed or other organic material can be subjected to decomposition processes thus reducing water quality and oxygen, compromising animal welfare during longer journey; sand poses the risk of H_2S release from fouled sediments.

4.4: Squid

The most recommended methods are nets, in particular Pound nets (**PN**) or Seine nets (**SN**) which are large enough to catch a reasonable number of adult animals without excessive constraint.[64, 65] Bongo nets (**BN**) are reported as suitable for hatchlings (**Hatch**).[33, 66]

Baited or Light Traps (**BLT**)[67, 68] can also be used as they are size selective (**SzS)** and employed for catching of adults (**Ad**).

Japanese baited pots (**JBP**) could be employed as well, as they are similar to traps and are size selective (**SzS**), so they can be adjusted in dimensions for catching both juveniles (**Juv**) and adults (**Ad**).[69] Squid jigs are widely employed with bait or with light lures and barbless hooks but they are not recommended for welfare issues (**W**).[70-72] Trawl is not recommended[34, 45] also considering welfare and environmental issues (**W**, **E**).

For transport the most suitable method are plastic bags depending on the size of the species. Few specimens can be included in the same bag, depending on its volume, the duration of the journey and on the size of the animals. Individual bags should be the recommendation, increasing the chance of survival (journey up to 20h[15]).

Boxes can be employed keeping the animals in similar conditions to bags and can be of different forms (buckets, **Bu**; coolers, **Co**; barrels, **Ba**), but can be considered more secure in terms of resistance to insults and vibration. Animals are reported to survive up to 8-11h.[15, 73]

Tanks are not frequently used, but are recommended for a large sample size (**LSz**; 20 specimens in a 60 x 90 cm fiberglass tanks filled of seawater to a depth of 30 cm[74]) or for larger species. Containers for holding bags (**Chb**) can be used also as to ensure that an appropriate temperature is maintained during transport.

4.5: Octopus

The most recommended method is pots, particularly those dark with narrow entrance and larger inside.[75, 76] A lid can be added (but not needed); octopuses have the natural tendency to search for a den and remaining inside. Females and potentially attached eggs should not be taken for conservation issues; as for above any deviation from the 'rule' has to be properly justified for research purpose.

Japanese baited pots (**JBP**) are also recommended as they combine the advantages of both pots (shelter) and traps (bait).[69] These are size selective (**SzS**) and might be adjusted in dimension for catching both juveniles (**Juv**) and adults (**Ad**). Nets, are mostly adopted for hatchlings (**Hatch**)[33, 77] but are not considered suitable for adults (**Ad**) which rarely get easily trapped in, unless caught with hand net by trained personnel performing SCUBA diving. Jigs are rarely used with baits, but are not recommended for welfare issues (**W**); trawl is not recommended[34, 45] also considering welfare and environmental issues (**W, E**).

For transport the most recommended method are large tanks for individual rearing (**I**). Can be used also as **Chb** to ensure that an appropriate temperature is maintained during transport. Under such circumstances animals are reported to survive up to 12h.[78] Boxes can be employed with similar modalities to bags; the same for containers of different forms (tubes, **Tu**; jars, **J**; creels; **Cr**) that may be accommodated into boxes.

Boxes can be used as Containers for holding bags (**Chb**) to ensure that an appropriate temperature is maintained during transport, and these can accommodate also the pot used for collection/capture.

Animals are reported to survive up to 24h in PVC tubes of 16 cm in diameter, located in a 200 L tank.[36] This approach has been utilized for aquaculture purposes and may be tested as suitable for scientific purposes by assessing experimentally welfare indicators and/or stress levels, thus to prove limited PSDLH.

Finally, plastic bags are suitable for every life stages according to the size of the animal. These can also be utilized for hatchlings (**Hatch**), even at high densities with high survival chances (at 6, 12, 24h).[36] For small body-size octopuses a survival of 8-10h has been reported[15] and if appropriately sealed (e.g., twisted at the top and folded over) and doubly secured, could survive over 12h.[14, 79]

Bags are appropriate for individual housing of sub-adult and adult specimens during transport, if adequate volume of seawater and oxygen is provided.

Suppl_5: Topics and Learning outcomes of an accredited course for collectors and transporters of live wild cephalopods to be used in scientific research

The content of the course we propose follows the modular scheme and organization included in "A working document on the development of a common education and training framework to fulfil the requirements under the Directive" Brussels, 19-20 February 2014 by the National Competent Authorities for the implementation of Directive 2010/63/EU on the protection of animals used for scientific purposes.

The training will be provided through the attendance of a 20 hours course, designed and delivered as part of the Cephalopod Biology and Care (CBC) FELASA accredited Training Program. The structure of the training program includes theoretical and practical sessions (at least 8 additional hours), around learning outcomes based on defined assessment and pass-fail criteria.

For collectors and transporters, the skills that the course should provide are suggested to be considered equivalent to Directive 2010/63/EU Functions A), C) and D).

General principles

Collectors, transporters and shippers should become familiar with some essential concepts which will be provided through a 20-hour training (spanned in three days). Attention will be given to practical aspects and hands-on-training aimed at getting the trainees more easily involved with the recommended, standardised, and validated equipment/protocols to be used during the actual capture and transport. Training will involve practical session to inform how to properly handle a given cephalopod species.

The practical skills will be the object of evaluation (OSPE) carried out after Trainees being successful in the first theoretical training phase.

Suppl_Box 1: Training Plan

Abbreviations included – **ATA**: Animal Transportation Association; **AWB**: Animal Welfare Body; **DV**: Designated Veterinarian; **IATA**: International Air Transport Association; **LAR**: Live Animal Regulation; **NCA**: National Competent Authority; **PSDLH**: Pain, Suffering, Distress and Lasting Harm; **TAC**: Total Allowable Catch.

Collectors	*Transporters*
**1. Sustainability, science and profit (theory)**	
1.1 Indicate how good welfare can promote good science<br>1.2 Describe how sustainability and animal welfare may influence experimental outcome<br>1.3 Recognize how applying sustainability and animal welfare principles may improve earnings<br>1.4 Explain why and how acquiring competence and specialization is crucial for both science and income.	1.1 Indicate how good welfare can promote good science<br>1.2 Describe how sustainability and animal welfare may influence experimental outcome<br>1.3 Recognize how applying sustainability and animal welfare principles may improve earnings<br>1.4 Explain why and how acquiring competence and specialization is crucial for both science and income.
**2. Legislations and guidelines for laboratory animals (theory)**	
2.1 Identify national and EU laws regulating the scientific use of laboratory animals (with particular attention to cephalopods as the sole invertebrate taxon)<br>2.2 Identify and describe related animal welfare legislation<br>2.3 Describe authorisation needed before acting as supplier of wild cephalopods<br>2.4 Describe the roles and responsibilities of the local AWB, NCA, DV for the protection of animals used for scientific purposes.	2.1 Identify national and EU laws regulating the scientific use of laboratory animals (with particular attention to cephalopods as the sole invertebrate taxon)<br>2.2 Identify and describe related animal welfare legislation<br>2.3 Describe authorisation needed before acting as supplier of wild cephalopods<br>2.4 Describe the roles and responsibilities of the local AWB, NCA, DV for the protection of animals used for scientific purposes.
**3. Ethic and animal welfare (theory)**	
3.1 Describe society views concerning scientific uses of animals and recognize the need to respect these<br>3.2 Describe the responsibility and the importance of having a respectful and humane attitude when dealing with animals in research<br>3.3 Identify ethical and animal welfare issues and their consequences during capture	3.1 Describe society views concerning scientific uses of animals and Recognize the need to respect these<br>3.2 Describe the responsibility and the importance of having a respectful and humane attitude when dealing with animals in research<br>3.3 Identify ethical and animal welfare issues and their consequences before/during transport

Collectors	*Transporters*
3.4 Describe the concepts of ThreeRs and Five Freedoms and how these apply to laboratory species.	3.4 Describe the concepts of ThreeRs and Five Freedoms and how these apply to laboratory species
**4. General and species-specific biology (theory + practice)**	
4.1 Describe basic anatomy, physiology, reproduction and behaviour of the most relevant cephalopod species<br>4.2 Describe dietary requirements of the most relevant cephalopod species and explain how these can be met<br>4.3 Indicate how modes of capture may influence animal welfare and physiology<br>4.4 Be able to approach, handle/pick up and restrain an animal in a humane way without causing PSDLH.	4.1 Describe basic anatomy, physiology, reproduction and behaviour of the most relevant cephalopod species<br>4.2 Describe dietary requirements of the most relevant cephalopod species and explain how these can be met<br>4.3 Indicate how modes of transport may influence animal welfare and physiology<br>4.4 Be able to approach, handle/pick up and restrain an animal in a humane way and put it in a proper container without causing PSDLH
**5. Harm-benefit and severity assessment (theory)**	
5.1 Recognize normal behaviour of the species in relation to its environment and physiological status<br>5.2 Describe the concept of PSDLH<br>5.3 Recognize abnormal behaviour and PSDLH<br>5.4 Define how to assess harm-benefit when doing research<br>5.5 Describe the meaning of procedures and the severity classification system<br>5.6 Describe how to assess the severity levels and give some examples.	5.1 Recognize normal behaviour of the species in relation to its environment and physiological status.<br>5.2 Describe the concept of PSDLH<br>5.3 Recognize abnormal behaviour and PSDLH<br>5.4 Define how to assess harm-benefit when doing research<br>5.5 Describe the meaning of procedures and the severity classification system<br>5.6 Describe how to assess the severity levels and give some examples
**6. Protocols for capture and transport (theory + practice)**	
6.1 Identify national, EU and intercontinental laws regulating capture and collection of live wild cephalopods (licenses, TAC)<br>6.2 Describe the available guidelines and good practices for the capture of live wild cephalopods<br>6.3 Recognize and describe any potential cause of suffering during capture, handling and collection	6.1 Identify national, EU and intercontinental laws regulating transport and shipment of live wild cephalopods<br>6.2 Describe the available guidelines for the transport of live wild cephalopods (e.g., IATA and ATA LAR)<br>6.3 Recognize and describe any potential cause of suffering during transport, handling and shipping

Collectors	*Transporters*
6.4 Identify and use the less traumatic capture gear for each target cephalopod species<br>6.5 Be able to choose and apply the best method according to the species and life stage	6.4 Identify the most appropriate container to transport each target cephalopod species<br>6.5 Be able to choose and apply the best method according to the species and life stage<br>6.6 Identify in advance all the requirements (e.g., documentations, hygiene, container, means of transport) according to the species and the duration of the journey
**7. Handling, sedation and humane killing (theory + practice)**	
7.1 Define sedation, local and general anaesthesia<br>7.2 Define balanced anaesthesia and indicate how to achieve an acceptable degree of unconsciousness<br>7.3 Relate why and when sedation or anaesthesia might be used for capture<br>7.4 Define methods for optimising post-anaesthetic recovery<br>7.5 Describe the principles of humane killing<br>7.6 Identify different methods for humanely kill and how to select the most appropriate method<br>7.7 Explain why someone competent to kill animals should be available at all times.	7.1 Define sedation, local and general anaesthesia<br>7.2 Define balanced anaesthesia and indicate how to achieve an acceptable degree of unconsciousness<br>7.3 Relate why and when sedation or anaesthesia might be used prior to transport<br>7.4 Define methods for optimising post-anaesthetic recovery<br>7.5 Describe the principles of humane killing<br>7.6 Identify different methods for humanely kill and how to select the most appropriate method<br>7.7 Explain why someone competent to kill animals should be always available.

References

1. Pieroni EM, Sykes A, Galligioni V, et al. Review on the methods of capture and transport of cephalopods for scientific purposes. *CephRes Publications and Reference Docs doi: 10.53124/cephres.202201* (2022, accessed September 2022).
2. Mouritsen OG and Styrbæk K. Cephalopod Gastronomy—A Promise for the Future. *Frontiers in Communication* 2018; 3. DOI: 10.3389/fcomm.2018.00038.
3. Ainsworth GB, Pita P, Garcia Rodrigues J, et al. Disentangling global market drivers for cephalopods to foster transformations towards sustainable seafood systems. *People and Nature* 2023; 5: 508-528. DOI: 10.1002/pan3.10442.
4. Globefish. World Congress on Cephalopods: Markets and Trade, http://www.fao.org/in-action/globefish/news-events/details-news/en/c/449821/ (2016, accessed January 2021).
5. Lishchenko F, Perales-Raya C, Barrett C, et al. A review of recent studies on the life history and ecology of European cephalopods with emphasis on species with the greatest commercial fishery and culture potential. *Fisheries Research* 2021; 236: 105847. DOI: https://doi.org/10.1016/j.fishres.2020.105847.
6. Olfert ED, Cross BM and McWilliam AA. *Guide to the care and use of experimental animals*. Canadian Council on Animal Care Ottawa, 1993.
7. European Parliament and Council of the European Union. Directive 2010/63/EU of the European Parliament and of the Council of 22 September 2010 on the protection of animals used for scientific purposes, (2010, https://eur-lex.europa.eu/legal-content/EN/ALL/?uri=CELEX:32010L0063).
8. UK Statutory Instruments and the Secretary of State. The Welfare of Animals (Transport) (England) Order 2006, https://www.legislation.gov.uk/uksi/2006/3260/introduction/made (2006).
9. Convention on International Trade in Endangered Species. Appendices I, II and III, https://cites.org/eng/app/appendices.php (2020, accessed March 2021).
10. National Research Council Committee for the Update of the Guide for the Care and Use of Laboratory Animals. The National Academies Collection: Reports funded by National Institutes of Health. *Guide for the Care and Use of Laboratory Animals*. Washington (DC): National Academies Press (US) Copyright © 2011, National Academy of Sciences., 2011.
11. National Health and Medical Research Council. *Australian code for the care and use of animals for scientific purposes 8th Edition*. Canberra: National Health and Medical Research Council, 2013.
12. Council of the European Union. Council Regulation (EC) No 1/2005 of 22 December 2004 on the protection of animals during transport and related operations and amending Directives 64/432/EEC and 93/119/EC and Regulation (EC) No 1255/97, (2004, accessed https://eur-lex.europa.eu/legal-content/en/ALL/?uri=CELEX%3A32005R0001).

13. Commission of the European Communities. Commission Recommendation of 18 June 2007 on guidelines for the accommodation and care of animals used for experimental and other scientific purposes (notified under document number C(2007) 2525), (2007, accessed https://eur-lex.europa.eu/legal-content/EN/TXT/?uri=CELEX%3A32007H0526).

14. Fiorito G, Affuso A, Basil J, et al. Guidelines for the Care and Welfare of Cephalopods in Research - A consensus based on an initiative by CephRes, FELASA and the Boyd Group. *Lab Anim* 2015; 49: 1-90.

15. Budelmann BU. Cephalopoda. *The UFAW handbook on the care and management of laboratory and other research animals* 2010: 787-817.

16. Grimpe G. Pflege, Behandlung und Zucht der Cephalopoden fur zoologische und physiologische Zwecke. In: Äberhalden E (ed) *Handbuch der biologischen Arbeitsmethoden*. Berlin, Wien: Verlag Urban & Schwarzenberg, 1928, pp.331-402.

17. De Sio F, Hanke FD, Warnke K, et al. E Pluribus Octo–Building Consensus on Standards of Care and Experimentation in Cephalopod Research; a Historical Outlook. *Frontiers in Physiology* 2020; 11: 645.

18. Boyle PR. *The UFAW handbook on the care and management of cephalopods in the laboratory*. Potters Bar Universities Federation for Animal Welfare, 1991, p.915.

19. Berka R. *The Transport of Live Fish: A Review*. Rome: European Inland Fisheries Advisory Commission (EIFAC); FAO - Food and Agriculture Organization of the United Nations, 1986.

20. Council of Europe. European Convention for the Protection of Animals during International Transport (Revised), (2003, accessed https://www.coe.int/en/web/conventions/full-list/-/conventions/rms/0900001680083710).

21. Swallow J, Anderson D, Buckwell AC, et al. Guidance on the transport of laboratory animals. *Lab Anim* 2005; 39: 1-39. DOI: 10.1258/0023677052886493.

22. Seibel BA, Robison BH and Haddock SHD. Post-spawning egg care by a squid. *Nature* 2005; 438: 929-929. DOI: 10.1038/438929a.

23. Bush SL, Hoving HJT, Huffard CL, et al. Brooding and sperm storage by the deep-sea squid *Bathyteuthis berryi* (Cephalopoda: Decapodiformes). *Journal of the Marine Biological Association of the United Kingdom* 2012; 92: 1629.

24. Joll LM. Mating, egg-laying and hatching of *Octopus tetricus* (Mollusca: Cephalopoda) in the laboratory. *Marine Biology* 1976; 36: 327-333. DOI: 10.1007/BF00389194.

25. Mangold K and von Boletzky S. New data on reproductive biology and growth of *Octopus vulgaris*. *Marine Biology* 1973; 19: 7-12. DOI: 10.1007/BF00355414.

26. Uriarte I, Espinoza V, Gutiérrez R, et al. Key aspects of egg incubation in Patagonian red octopus (*Enteroctopus megalocyathus*) for cultivation purposes. *Aquaculture* 2014; 424-425: 158-166. DOI: https://doi.org/10.1016/j.aquaculture.2013.12.039.

27. Mangold K. *Octopus vulgaris*. In: Boyle PR (ed) *Cephalopod Life Cycles Species Accounts*. London: Academic Press, 1983, pp.335-364.

28. Deryckere A, Styfhals R, Vidal EAG, et al. A practical staging atlas to study embryonic development of *Octopus vulgaris* under controlled laboratory conditions. *BMC Developmental Biology* 2020; 20: 7. DOI: 10.1186/s12861-020-00212-6.

29. Spreitzenbarth S and Jeffs A. Egg survival and morphometric development of a merobenthic octopus, *Octopus tetricus*, embryos in an artificial octopus egg rearing system. *Aquaculture* 2020; 526: 735389. DOI: https://doi.org/10.1016/j.aquaculture.2020.735389.

30. Villanueva R. Experimental rearing and growth of planktonic Octopus vulgaris from hatching to settlement. *Canadian Journal of Fisheries and Aquatic Sciences* 1995; 52: 2639-2650.

31. Boletzky Sv. Réflexions sur les stratégies de reproduction chez les Céphalopodes. *Bull Soc Zool Fr* 1981; 106: 293-304.

32. Boletzky SV. Recent studies on spawning, embryonic development, and hatching in the Cephalopoda. *Advances in Marine Biology*. Elsevier, 1989, pp.85-115.

33. Otero J, Álvarez-Salgado XA, González ÁF, et al. Wind-driven upwelling effects on cephalopod paralarvae: *Octopus vulgaris* and Loliginidae off the Galician coast (NE Atlantic). *Progress in Oceanography* 2016; 141: 130-143.

34. Boletzky SV and Hanlon RT. A Review of the Laboratory Maintenance, Rearing and Culture of Cephalopod Molluscs. *Memoirs of the National Museum of Victoria* 1983; 44: 147-186.

35. Boletzky Sv. Biology of early life stages in cephalopod molluscs. *Advances in Marine Biology* 2003; 44: 143-203.

36. Fuentes L, Iglesias J, Sánchez FJ, et al. Métodos de transporte de paralarvas y adultos de pulpo *Octopus vulgaris* Cuvier, 1797. *Boletín Instituto Español de Oceanografía* 2011; 21: 155-162.

37. Sykes AV, Almansa E, Cooke GM, et al. The digestive tract of cephalopods: a neglected topic of relevance to animal welfare in the laboratory and aquaculture. *Frontiers in Physiology* 2017; 8: 492.

38. Hanlon RT. Maintenance, rearing, and culture of teuthoid and sepioid squids. *Squid as experimental animals*. Springer, 1990, pp.35-62.

39. Jones JB. Environmental impact of trawling on the seabed: A review. *New Zealand Journal of Marine and Freshwater Research* 1992; 26: 59-67. DOI: 10.1080/00288330.1992.9516500.

40. Johnson AF, Gorelli G, Jenkins SR, et al. Effects of bottom trawling on fish foraging and feeding. *Proceedings of the Royal Society B: Biological Sciences* 2015; 282: 20142336. DOI: doi:10.1098/rspb.2014.2336.

41. Good E, Holman LE, Pusceddu A, et al. Detection of community-wide impacts of bottom trawl fishing on deep-sea assemblages using environmental DNA

metabarcoding. *Marine Pollution Bulletin* 2022; 183: 114062. DOI: https://doi.org/10.1016/j.marpolbul.2022.114062.

42. Gray JS, Dayton P, Thrush S, et al. On effects of trawling, benthos and sampling design. *Marine Pollution Bulletin* 2006; 52: 840-843. DOI: https://doi.org/10.1016/j.marpolbul.2006.07.003.
43. Hiddink JG, Jennings S, Sciberras M, et al. Global analysis of depletion and recovery of seabed biota after bottom trawling disturbance. *Proceedings of the National Academy of Sciences* 2017; 114: 8301-8306. DOI: doi:10.1073/pnas.1618858114.
44. van Denderen PD, Bolam SG, Hiddink JG, et al. Similar effects of bottom trawling and natural disturbance on composition and function of benthic communities across habitats. *Marine Ecology Progress Series* 2015; 541: 31-43.
45. Rathjen WF. Cephalopod capture methods: an overview. *Bulletin of Marine Science* 1991; 49: 494-505.
46. O'Dor RK, Wells J and Wells MJ. Speed, jet pressure and oxygen consumption relationships in free-swimming *Nautilus*. *Journal of Experimental Biology* 1990; 154: 383-396.
47. Carlson BA. Collection and aquarium maintenance of *Nautilus*. In: N.H SWBL (ed) *Nautilus*. Springer, 2010, 1991, pp.563-578.
48. Oba T, Kai M and Tanabe K. Early life history and habitat of *Nautilus pompilius* inferred from oxygen isotope examinations. *Marine Biology* 1992; 113: 211-217.
49. Uchiyama K and Tanabe K. Hatching of *Nautilus macromphalus* in the Toba aquarium, Japan. *Advancing research on living and fossil cephalopods*. Springer, 1999, pp.13-16.
50. Dunstan A, Bradshaw CJA and Marshall J. Nautilus at risk–estimating population size and demography of *Nautilus pompilius*. *PloS One* 2011; 6: e16716.
51. Linzmeier BJ, Kozdon R, Peters SE, et al. Oxygen isotope variability within *Nautilus* shell growth bands. *PLoS One* 2016; 11: e0153890.
52. Muntz WRA. Effects of light on the efficacy of traps for Nautilus pompilius. *Marine Behaviour and Physiology* 1994; 24: 189-193.
53. Watanuki N and Kawamura G. A review of cuttlefish basket trap fishery. *South Pacific Study* 1999; 19: 31-48.
54. Moltschaniwskyj NA, Hall K, Lipinski MR, et al. Ethical and welfare considerations when using cephalopods as experimental animals. *Reviews in Fish Biology and Fisheries* 2007; 17: 455-476.
55. Solé M, Monge M, André M, et al. A proteomic analysis of the statocyst endolymph in common cuttlefish (*Sepia officinalis*): an assessment of acoustic trauma after exposure to sound. *Scientific reports* 2019; 9: 1-12.
56. O'Brien CE, Bellanger C, Jozet-Alves C, et al. Stressful conditions affect reproducing cuttlefish (*Sepia officinalis*), reducing egg output and quality. *ICES Journal of Marine Science* 2018; 75: 2060-2069.

57. Pereira F, Vasconcelos P, Moreno A, et al. Catches of *Sepia officinalis* in the small-scale cuttlefish trap fishery off the Algarve coast (southern Portugal). *Fisheries Research* 2019; 214: 117-125.

58. Şen H. Effects of String diameter preference of *Sepia officinalis* (L. 1758) during spawning in captivity. *Journal of Fisheries Sciences* 2013; 7: 297-301.

59. Wei SL and Young RE. Development of symbiotic bacterial bioluminescence in a nearshore cephalopod, *Euprymna scolopes*. *Marine Biology* 1989; 103: 541-546.

60. Montgomery MK and McFall-Ngai M. Embryonic development of the light organ of the sepiolid squid *Euprymna scolopes* Berry. *The Biological Bulletin* 1993; 184: 296-308.

61. Jones NJE, Ridgway ID and Richardson CA. Transport of cuttlefish, *Sepia officinalis*, eggs under dry and damp conditions. *Journal of Molluscan Studies* 2009; 75: 192-194.

62. Nabhitabhata J and Nishiguchi MK. Euprymna hyllebergi and Euprymna tasmanica. *Cephalopod Culture*. Springer, 2014, pp.253-269.

63. Hanlon RT, Claes MF, Ashcraft SE, et al. Laboratory culture of the sepiolid squid *Euprymna scolopes*: a model system for bacteria-animal symbiosis. *The Biological Bulletin* 1997; 192: 364-374.

64. Chabala LD, Morello RS, Busath D, et al. Capture, transport, and maintenance of live squid (*Loligo pealei*) for electrophysiological studies. *Pflügers Archiv* 1986; 407: 105-108.

65. Gonçalves JM, Porteiro FM, Cardigos F, et al. The Azorean *Loligo forbesi* (Cephalopoda: Loliginidae) in captivity: transport, handling, maintenance, tagging and survival. *Marine Biodiversity Records* 2009; 2: e120.

66. Olmos-Pérez L, Pierce GJ, Roura Á, et al. Barcoding and morphometry to identify and assess genetic population differentiation and size variability in loliginid squid paralarvae from NE Atlantic (Spain). *Marine Biology* 2018; 165: 136.

67. Thorrold SR. Evaluating the performance of light traps for sampling small fish and squid in open waters of the central Great Barrier Reef lagoon. *Marine Ecology Progress Series* 1992; 89: 277-285.

68. Vidal EAG, Villanueva R, Andrade JP, et al. Chapter One - Cephalopod Culture: Current Status of Main Biological Models and Research Priorities. In: Vidal EAG (ed) *Advances in Marine Biology*. London, UK: Academic Press, 2014, pp.1-98.

69. Carreira GP and Gonçalves JM. Catching *Octopus vulgaris* with traps in the Azores: first trials employing Japanese baited pots in the Atlantic. *Marine Biodiversity Records* 2009; 2: e114.

70. Flores EEC, Igarashi S, Mikami T, et al. Studies on Squid Behavior in Relation to Fishing: I. On the handling of squid, *Todarodes pacificus* Steenstrup, for behavioral study. *Bulletin of the Faculty of Fisheries Hokkaido University* 1976; 27: 145-151.

71. Cabanellas-Reboredo M, Alós J, Palmer M, et al. Simulating the indirect handline jigging effects on the European squid *Loligo vulgaris* in captivity. *Fisheries Research* 2011; 110: 435-440.

72. Perretti CT, Zerofski PJ and Sedarat M. The spawning dynamics of California market squid (*Doryteuthis opalescens*) as revealed by laboratory observations. *Journal of Molluscan Studies* 2016; 82: 37-42.

73. LaRoe ET. The culture and maintenance of the loliginid squids Sepioteuthis sepioidea and *Doryteuthis plei*. *Marine Biology* 1971; 9: 9-25.

74. O'Dor RK, Durward RD and Balch N. Maintenance and maturation of squid (*Illex illecebrosus*) in a 15 meter circular pool. *The Biological Bulletin* 1977; 153: 322-335.

75. Borges TC, Calixto P and Sendão J. The common octopus fishery in South Portugal: a new shelter-pot. *Mediterránea Serie de Estudios Biológico* 2015: 130-154. DOI: 10.14198/MDTRRA2015.ESP.07.

76. Walker JJ, Longo N and Bitterman ME. The octopus in the laboratory. Handling, maintenance, training. *Behav Res Methods Instrum* 1970; 2: 15-18.

77. Castellano GC, da Veiga MPT, Mazzini FS, et al. Paralarvae of *Octopus vulgaris* Type II are stenohaline conformers: relationship to field distribution and dispersal. *Hydrobiologia* 2018; 808: 71-82.

78. Boyle PR. Methods for the aquarium maintenance of the common octopus of British waters, *Eledone cirrhosa*. *Laboratory Animals* 1981; 15: 327-331.

79. Fiorito G, Affuso A, Anderson DB, et al. Cephalopods in neuroscience: Regulations, Research and the 3Rs. *Invert Neurosci* 2014; 14: 13-36.
